# Structural, Computational, and Biomolecular Interaction Study of Europium(III) and Iron(III) Complexes with Pyridoxal-Semicarbazone Ligand

**DOI:** 10.3390/ijms26115289

**Published:** 2025-05-30

**Authors:** Violeta Jevtovic, Stefan Perendija, Aljazi Abdullah Alrashidi, Maha Awjan Alreshidi, Elham A. Alzahrani, Odeh A. O. Alshammari, Mostafa Aly Hussien, Jasmina Dimitrić Marković, Dušan Dimić

**Affiliations:** 1 Department of Chemistry, College of Science, University of Ha’il, Ha’il 81451, Saudi Arabia; 2 Faculty of Physical Chemistry, University of Belgrade, Studentski Trg 12-16, 11158 Belgrade, Serbia; 3 Department of Chemistry, Faculty of Science, King Abdul Aziz University, Jeddah 21589, Saudi Arabia

**Keywords:** pyridoxal-semicarbazone, europium(III), iron(III), QTAIM, DFT, HSA, CT-DNA, molecular docking, crystallography

## Abstract

The coordination chemistry, structural characterization, and biomolecular interactions of europium(III) and iron(III) complexes with the pyridoxal-semicarbazone (PLSC) ligand were thoroughly examined using experimental and computational approaches. Single-crystal X-ray diffraction revealed that the europium complex exhibits a nine-coordinate geometry with one protonated and one deprotonated PLSC ligand and nitrato and aqua ligands. In contrast, the iron complex adopts a six-coordinate structure featuring a monoprotonated PLSC, two chlorido, and an aqua ligand. Hirshfeld surface analysis confirmed the significance of intermolecular contacts in stabilizing the crystal lattice. Theoretical geometry optimizations using DFT methods demonstrated excellent agreement with experimental bond lengths and angles, thereby validating the reliability of the chosen computational levels for subsequent quantum chemical analyses. Quantum Theory of Atoms in Molecules (QTAIM) analysis was employed to investigate the nature of metal–ligand interactions, with variations based on the identity of the donor atom and the ligand’s protonation state. The biological potential of the complexes was evaluated through spectrofluorimetric titration and molecular docking. **Eu-PLSC** displayed stronger binding to human serum albumin (HSA), while **Fe-PLSC** showed higher affinity for calf thymus DNA (CT-DNA), driven by intercalation. Thermodynamic data confirmed spontaneous and enthalpy-driven interactions. These findings support using PLSC-based metal complexes as promising candidates for future biomedical applications, particularly in drug delivery and DNA targeting.

## 1. Introduction

Cancer is a genetic disorder resulting from alterations in genes that regulate cellular functions, particularly those involved in growth and division [1]. Despite advancements in early diagnosis and treatment, many tumor types remain resistant to currently available therapies. Cisplatin has demonstrated anticancer effects in clinical practice, paving the way for the introduction of transition metal complexes in medicine. The interaction of cisplatin with the DNA molecules of tumor cells is the primary mechanism of its activity [2,3]. However, the limited selectivity, high toxicity, and development of resistance have driven the continued pursuit of novel metal-based chemotherapeutic agents.

The semicarbazide derivatives of the general formula R–CH=N–NH–CX–NH_2_, named semicarbazones, are formed in the reaction between semicarbazide and aldehyde/ketones [4]. These compounds can also be described as a sub-class of Schiff bases, as the imine functional group is present. Due to their specific structural features, these ligands can be unidentate, bidentate, and multidentate, depending on the aldehyde/ketone used for synthesis [5]. The protonation/deprotonation of ligands also influences the stability and structural features of the resulting complexes. Many transition metal complexes containing semicarbazones have been described in the literature due to their stability, structural versatility, chelating properties, and intense color [6,7,8]. Pyridoxal-semicarbazone (PLSC), used as a ligand in this contribution, is obtained from pyridoxal and semicarbazone [9]. The structure of PLSC features three donor atoms: a phenolic oxygen, an azomethine nitrogen, and a carbonyl oxygen. The monoanionic, dianionic, and neutral form of the ligand are known, as explained in reference [10]. The neutral form contains protonated pyridine and azomethine nitrogen sites, while the monoanionic form has deprotonated pyridine at a pH above 7.

The rare earth elements, including europium, have unique properties due to the presence of 4f-orbitals and different oxidation states [11,12]. Their coordination compounds are usually characterized by the presence of six to twelve metal–ligand bonds [11,13]. This variety of structural features enables their potential use in various applications, such as technical materials, optoelectronics, biomedicine, and counterfeiting [14,15,16,17,18]. The investigation of complexes garners interest due to distinctive features such as highly specific emission bands, prolonged excited-state lifetimes, and large Stokes shifts, among others [19,20]. The change in ligands and co-ligands allows a subtle change in biological activity and represents a method for further application. Felício and coworkers prepared mononuclear Eu and bimetallic Eu/Cu complexes, and higher activity towards promoting cell death was found for the latter complex, while the fluorescence emission was higher in the mononuclear complex [21]. Rocha and coworkers described the synthesis, characterization, and interactions with biomolecules of a nine-coordinated europium(III) complex containing the lawsone ligand [22]. The antioxidant and anticancer properties of the Eu(III) complex with the Schiff base ligand (2,2′-(thiophene-2,5-diylbis(methaneylyl-idene))bis(azaneylyl-idene))diphenol were assessed in reference [23]. Mosbah and coworkers examined the cytotoxicity of the Eu(III) complex with a fenamic acid-based ligand towards MDA-MB-231 breast cancer cells [24]. Other Eu-Schiff base complexes are promising targets for the synthesis of new compounds due to their intriguing coordination behavior and wide-ranging applications in chemical, pharmaceutical, and industrial fields [25,26,27]. The interactions between Eu(III)-containing complexes with biological models, such as human serum albumin (HSA) and calf thymus DNA (CT-DNA), were described in references [22,23].

The synthesis of PLSC complexes with Fe(III), namely [Fe(PLSC)Cl_2_(H_2_O)]Cl and [Fe(PLSC-H)_2_]Cl·4H_2_O, was described in the paper by Jevtovic and coworkers in [28], although the crystallographic structures were not solved. The complexes containing iron(III) and the PLSC ligand were used as catalysts for the hydrogen evolution reaction [29]. The crystallographic structures and thermal characterization of [Fe^II^(PLSC)(H_2_O)SO_4_] and [Fe^III^(PLSC)(H_2_O)Cl_2_]Cl were reported, although with some limitations, in reference [30]. The crystallographic structure and antibacterial activity of [Fe^II^(PLSC)_2_](NO_3_)_2_·H_2_O were examined in [31] and compared to similar [Co(PLITSC-2H)(PLITSC-H)]·CH_3_OH (PLITSC is a pyridoxal S-methylisothiosemicarbazone ligand). A review of other metal complexes with pyridoxal semicarbazone-type ligands is presented in [32]. The quantum-chemical analysis and interactions with biomolecules of these complexes were not investigated in the available literature.

The aim of this contribution is to investigate and compare the coordination behavior, geometrical features, and ligand-binding modes of europium(III) and iron(III) complexes with the PLSC ligand using crystallographic, spectroscopic, and computational methods. Although the complexes differ in their counter ions (nitrate vs. chloride), both were selected to explore how different metal centers—representing a lanthanide and a transition metal—affect the structural and chemical properties of PLSC-based coordination compounds. The structural features of [Eu(PLSC)(PLSC-H)(NO_3_)(H_2_O)]·[NO_3_]·3(H_2_O) and [Fe(PLSC)Cl_2_(H_2_O)][Cl] were determined by X-ray diffraction and FTIR analyses. Hirshfeld surface analysis was employed to examine the intramolecular interactions within the crystal structure, emphasizing the importance of geometry and ligands. Density functional theory (DFT) methods were applied to optimize experimental structures, and the applicability of the selected level of theory was determined by comparing the experimental and theoretical bond lengths and angles. The Quantum Theory of Atoms in Molecules (QTAIM) was applied to examine the stability of complexes, particularly the metal–ligand interactions. The interactions of complexes with HSA and DNA were monitored using spectrofluorimetric titration and molecular docking simulations. The most probable positions for these interactions were determined.

## 2. Results and Discussion

### 2.1. Crystallographic Structural Analysis

**Figure 1:**
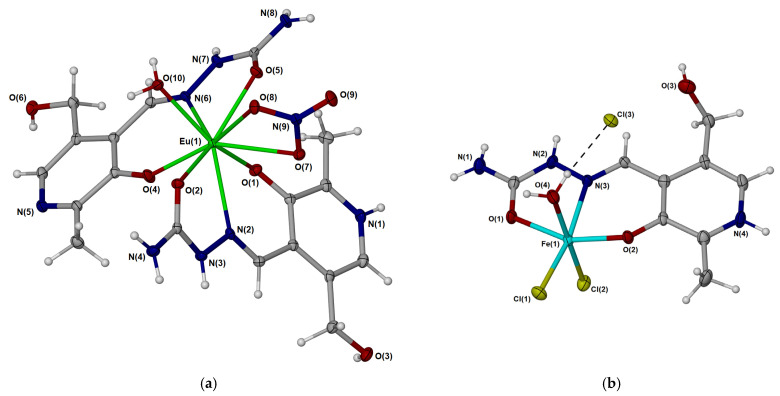
Molecular diagram of (**a**) [Eu(PLSC)(PLSC-H)(NO_3_)(H_2_O)]·[NO_3_]·3(H_2_O) and (**b**) [Fe(PLSC)Cl_2_(H_2_O)][Cl] with non-hydrogen atoms represented by 50% displacement ellipsoids and hydrogen atoms as spheres of arbitrary size. The uncoordinated [NO_3_]^−^ anion and water molecules in (**a**) have been omitted for clarity.

The complex containing europium(III) ions was prepared from the mixture of Eu(NO_3_)_2_ and the pyridoxal-semicarbazone ligand. The yellow crystals were examined by X-ray crystallographic analysis, and the structure presented in Figure 1 was obtained. The compound crystallizes in the triclinic space group *P_−1_*. This interesting nine-coordinated structure, containing ligands and anions, was found. In the structure, europium(III) ions are coordinated by two PLSC ligands, one in the protonated and the other in the deprotonated form. PLSC acts as a tridentate ligand, forming interactions through the azomethine nitrogen, phenyl oxygen, and carbonyl oxygen atoms. Two PLSC ligands differ in the presence of a hydrogen atom on the pyridine nitrogen. It is important to note that the bond lengths between donor atoms and the central metal ion do not depend significantly on the protonation of the PLSC ligand, as listed in Table S1. The shortest bonds were found between Eu(III) and phenyl oxygen atoms (2.22 and 2.28 Å), which is expected due to the extended delocalization throughout the phenyl ring. The bond lengths between carbonyl oxygen and the central metal ion are slightly longer, around 2.42 Å. When azomethine nitrogen atoms are included, the bond lengths are 2.62 Å. A similar situation arises when considering the angles between donor atoms in PLSC. Complex **Eu-PLSC** also contains a doubly coordinated nitrate ion, with two bonds formed between oxygen atoms and the central metal ion. These bonds have bond lengths of 2.58 and 2.63 Å. The coordination induces elongation of the respective N−O bonds of the nitrate anion to 1.28/1.27 Å compared to the non-bonded oxygen atom (1.22 Å). The angle between oxygen atoms of the nitrato ligand is also lower compared to the angles including the non-bonded oxygen atom (115.9 vs. 122.4/121.7°, Table S2). **Eu-PLSC** contains the aqua ligand, with the Eu−O bond length being 2.43 Å. It should be noted that these characteristics of Eu−O bonds in this complex greatly depend on the characteristics of the other atoms surrounding oxygen, and this will be examined in detail in the QTAIM analysis later in this paper. In the outer sphere, **Eu-PLSC** has one nitrate counterion and three co-crystallized water molecules. Complete lists of bond lengths and angles are given in Tables S1 and S2 in the Supplementary Materials. The bonds that include hydrogen atoms were omitted from the comparison, as hydrogen atoms bonded to carbon were placed based on a riding model, while the positions of hydrogen atoms bonded to oxygen and nitrogen were derived from the difference Fourier map.

**Figure 2:**
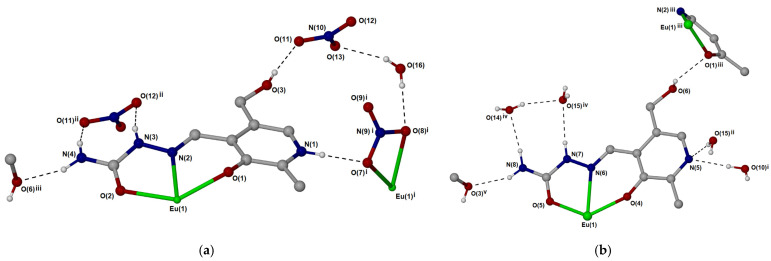
Hydrogen bonding in [Eu(PLSC)(PLSC-H)(NO_3_)(H_2_O)]·[NO_3_]·3(H_2_O). (**a**) Hydrogen bonding involving ligand 1. Atoms generated by crystallographic symmetry: i 1-x,1-y,1-z; ii 1+x,y,z; iii 2-x,-y,2-z. (**b**) Hydrogen bonding involving ligand 2. Atoms generated by crystallographic symmetry: i 2-x,-y,2-z; ii x,y-1,z; iii 1-x,-y,2-z; iv 1-x,1-y,2-z; v 1-x,1-y,1-z. Hydrogen bonds are listed in Table S3.

The crystallographic structure of the **Eu-PLSC** complex is additionally stabilized by the presence of several hydrogen bonds, as depicted in Figure 2, Figures S1 and S2. The characteristics of these bonds are shown in Table S3. The oxygen atom of the hydroxymethyl group forms a hydrogen bond with the nitrate anion with a bond length of 2.05 Å. Additionally, two PLSC ligands from two different units form an interaction between the hydroxymethyl group and the phenyl oxygen (2.30 Å, Figure 2). The co-crystalized water molecules interact with polar groups of the PLSC ligand, for example, with the deprotonated nitrate atom of the pyridine moiety (1.99 and 2.21 Å). The **Eu-PLSC** complex is also stabilized by the hydrogen bonds in which the PLSC ligand acts as a proton donor through protonated nitrogen atoms of the ending amino group or aliphatic chain, while water molecules are proton acceptors. The same type of interaction can be found between protonated nitrogen atoms and nitrate anion (2.10 and 2.43 Å). Two nitrate anions are bridged by one water molecule, which forms hydrogen bonds in which the water molecule is a proton donor, with bond lengths of 2.05 and 2.09 Å.

The second complex was formed from the mixture of the PLSC ligand and FeCl_3_·6H_2_O. The red-orange crystals were examined, and it was found that the compound crystallized in an orthorhombic system with the *P2_1_2_1_2_1_* space group. The crystallographic structure of [Fe(PLSC)Cl_2_(H_2_O)][Cl] is given in Figure 1, similar to the one obtained in [30]. During synthesis, only one crystalline product was obtained (Figure S3). As shown in the figure, the structure is significantly simpler than the one previously discussed. It contains a protonated PLSC ligand that forms coordination bonds with the central metal ion through three donor atoms. As expected from the previous compound, the shortest bond is formed between the phenyl oxygen atom and Fe(III) with a bond length of 1.92 Å, followed by the bonds that include carbonyl oxygen (2.06 Å) and azomethine nitrogen (2.19 Å). These bonds are shorter than those in **Eu-PLSC**, although one of the reasons could be the presence of the second PLSC ligand in the latter structure, which leads to the repulsion of ligands and elongation of bonds. The inner sphere of the **Fe-PLSC** complex contains two chlorido ligands with bond lengths of 2.27 and 2.28 Å. There is also an aqua ligand coordinated to the central metal ion. The Fe−O bond length is 2.11 Å. This bond is shorter than the respective Eu−O bond. The QTAIM analysis was later performed to compare various factors influencing the coordination abilities of europium(III) and iron(III) in these compounds. The **Fe-PLSC** complex is neutralized by the chloride anion in the outer sphere. The remaining bond lengths and angles are listed in Tables S4 and S5.

**Figure 3:**
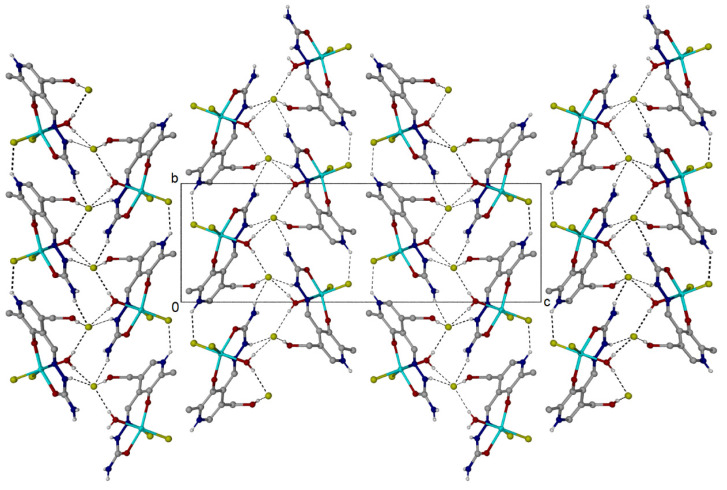
Cell contents of [Fe(PLSC)Cl_2_(H_2_O)][Cl] as viewed down the *a* axis. The structure comprises 2-D sheets parallel to the *ab* plane formed from N-H···Cl and O-H···Cl hydrogen bonds to the lattice [Cl]^−^ anion. An additional intermolecular N-H···Cl hydrogen bond exists between the pyridinium N-H and one of the coordinated Cl atoms.

The stabilization of **Fe-PLSC** structures also results from the hydrogen bond interactions, although their number and versatility are significantly lower (Figure 3 and Table S6). These interactions exist between the chloride anion and protonated pyridine nitrogen, the oxygen from the phenyl group, and the coordinated aqua ligand. The bond lengths are in a wide range between 2.21 and 2.85 Å, while the bond angles are between 141 and 175°. The interaction between protonated pyridine nitrogen and the chlorido ligand of two different units is present, with a bond length of 2.37 Å and an angle of 141° (Table S6). The value of the angle is a consequence of the steric hindrance and crystallographic structure rigidity.

### 2.2. Spectroscopic FTIR Analysis

The FTIR spectra of both complexes were recorded using the KBr pellet technique, and they are shown in Figures S4 and S5. In the region above 2500 cm^−1^, both complexes exhibit a broad peak due to the presence of water molecules in the structure, either as aqua ligands or co-crystallized solvent molecules. The peak between 3300 and 2500 cm^−1^ is significantly more intense in the case of **Eu-PLSC**, as three water molecules are integral to the crystal structure. Both complexes have a sharp peak assigned to the pyridine O−H stretching vibration (3271 cm^−1^ (**Eu-PLSC**) and 3463 cm^−1^ (**Fe-PLSC**)), as observed for other PLSC complexes [33]. The intense bands also contain N−H stretching vibrations that form hydrogen bonds that lead to the additional widening of the bands [34,35]. The protonated pyridine N−H stretching vibration is present at around 3100 (**Eu-PLSC**) and 3200 (**Fe-PLSC**) cm^−1^, similar to the copper-containing PLSC complex [10]. The peaks at 2887 (**Eu-PLSC**) and 2829 (**Fe-PLSC**) are attributed to the ^+^N−H vibration of the protonated PLSC ligand, as discussed in [36]. The contribution of C−H vibrations is not high, as there is a limited number of these bonds. The sp^3^ C−H bond vibrations are present below 3000 cm^−1^, while for sp^2^ carbon atoms, these bonds are assigned to bands above 3000 cm^−1^. The intensity of these bands also reflects the number of PLSC ligands in structures.

In the structure of the pure PLSC ligand, the most notable band at 1680 cm^−1^ is assigned to the C=O vibration [36]. Two bands at 1692 and 1615 cm^−1^ are assigned to this type of vibration in the spectrum of **Eu-PLSC**, while only one at 1689 cm^−1^ is in the spectrum of **Fe-PLSC**. These medium-intensity bands are a consequence of the interactions between carbonyl oxygen and central metal ions, as discussed in [10,36]. The spectrum of C=N stretching vibration in the PLSC ligand is positioned at 1570 cm^−1^ [33]. The same band in the spectrum of complexes is shifted towards lower wavenumbers (1538 cm^−1^ (**Eu-PLSC**) and 1568 cm^−1^ (**Fe-PLSC**)), indicating that this group is also part of the inner sphere of the complexes [36,37]. A similar shift in the azomethine functional group was observed in the Fe(III)-isoniazid hydrazone Schiff base complex [38]. The asymmetric stretching vibration of nitrate groups is assigned to the band at 1230 cm^−1^ [10]. The phenolic C−O group in the spectrum of the pure ligand is located at 1150 cm^−1^ [39], and in spectra of complexes is at 1159 (**Eu-PLSC**) and 1154 cm^−1^ (**Fe-PLSC**). Both complexes also have a C−O stretching vibration band at around 1000 cm^−1^. The region below 1000 cm^−1^ is characterized by various out-of-plane and torsional vibrations. Bands around 900 cm^−1^ are assigned to the bending vibrations of nitrate ions in the **Eu-PLSC** structure, similar to other complexes in the literature [10].

### 2.3. Hirshfeld Surface Analysis

**Figure 4:**
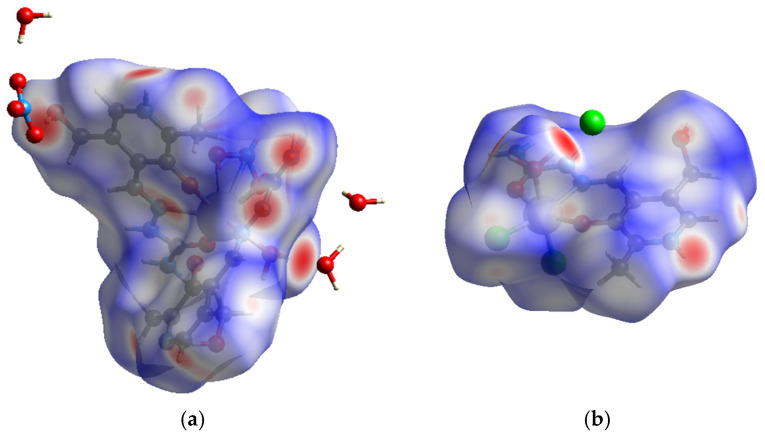
The Hirshfeld surface maps of (**a**) [Eu(PLSC)(PLSC-H)(NO_3_)(H_2_O)]^+^ and (**b**) [Fe(PLSC)Cl_2_(H_2_O)][Cl].

The crystallographic structures of two newly obtained compounds were subjected to the Hirshfeld surface analysis to examine intermolecular contacts that are important for the overall stability. The Hirshfeld surfaces of these compounds are shown in Figure 4, while the fingerprint plots of the most numerous contacts are given in Figures S6 and S7. In the case of **Eu-PLSC**, the most numerous contacts include H···O with a percentage of 40.1%. This is expected, as the structure of the complex contains oxygen atoms in aqua and nitrato ligands and a hydroxymethyl group, whereas the structures of the protonated and deprotonated PLSC ligands are abundant in hydrogen atoms. As previously discussed, the crystallographic structure of **Eu-PLSC** contains a large number of hydrogen bonds with co-crystallized solvent molecules. As the nitrogen atoms are also present in the amino group, aliphatic chain, and pyridine ring, contacts denoted as H···N contribute by 6.8%. The structure of PLSC also influences a high percentage of H···C contacts (14.2%). These bonds are weak and include interactions between the π-electron cloud and positively charged hydrogen atoms. Due to the complex geometry, the interactions containing europium(III) ions were not found, while contacts between two non-hydrogen atoms are present in a low percentage. These values are 1.6, 2.7, and 2.1% for the O···O, O···N, and O···C contacts, respectively. The weakest interactions are denoted as H···H, with a total percentage of 29.9%. These interactions occur between hydrogen atoms of different partial charges, resulting from various heteroatoms and delocalized electron clouds over ring structures and aliphatic chains.

**Figure 5:**
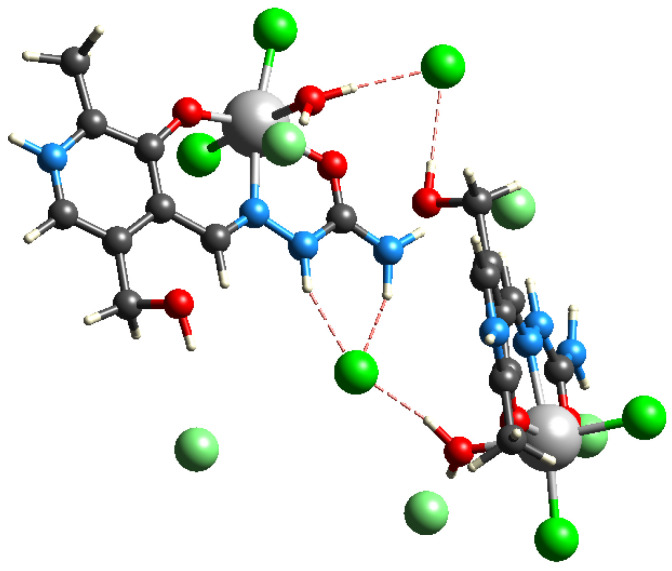
Cl···H contacts in the crystallographic structure of [Fe(PLSC)Cl_2_(H_2_O)][Cl].

The most numerous contacts in the crystallographic structure of **Fe-PLSC** contain chlorine atoms due to their abundance. In the first place, there are Cl···H contacts with a percentage of 38.1. These interactions include contacts between the chlorido ligands of one molecule and the hydrogen atoms of the PLSC ligand of the other. Additionally, interactions between the hydrogen atoms of PLSC are influenced by the counteranion present in the structure. The interactions between two chlorine atoms account for 0.7%; some of these contacts are depicted in Figure 5. The nitrogen and carbon atoms also form contacts with chlorine, characterized by 1.4% and 1.5%, respectively. The presence of water molecules is also important for the overall stabilization, as oxygen atoms interact with neighboring hydrogen atoms through O···H contacts (13.9%). The nitrogen atoms of the aliphatic chain and the amino group also interact with hydrogen atoms (3.8%). The interactions between hydrogen and carbon atoms are present in 11.4%, which is similar to those in the previously discussed compound. Weak interactions between hydrogen atoms are included in 25% of contacts. Polar groups of PLSC and the aqua ligand are the ones participating in these interactions. Other important contacts are presented in the fingerprint plots (Figure S5).

### 2.4. Theoretical Structural Analysis and Intramolecular Interactions

**Figure 6:**
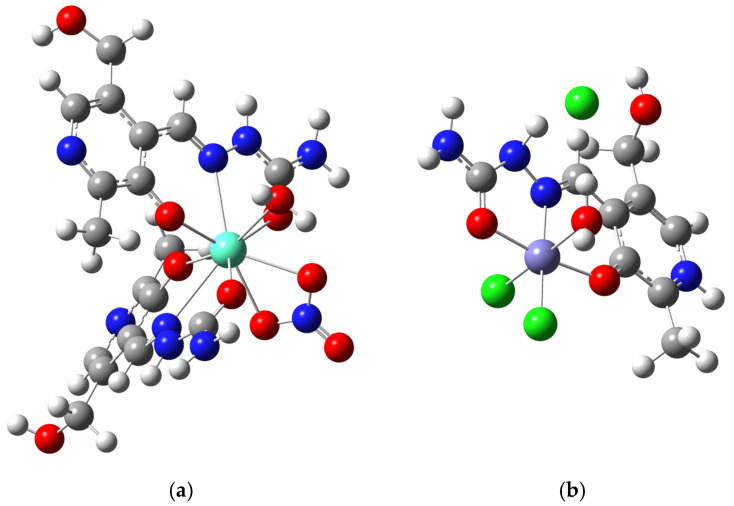
Optimized structures of (**a**) [Eu(PLSC)(PLSC-H)(NO_3_)(H_2_O)]^+^ (at B3LYP/6-31+G(d,p)(H,C,N,O)/def2-TZVP(Eu) level of theory) and (**b**) [Fe(PLSC)Cl_2_(H_2_O)][Cl] (at B3LYP/6-311++G(d,p)(H,C,N,O,Cl)/def2-TZVP(Fe) level of theory).

The crystallographic structures were used as the starting structures for optimization. Depending on the system, different levels of theory were chosen to facilitate optimization and further theoretical analysis of the interactions. Optimized structures are shown in Figure 6. For the **Eu-PLSC** complex, the B3LYP functional was selected along with a 6-31+G(d,p) basis set for H, C, N, and O and a def2-TZVP basis set for Eu. This same level of theory for europium-containing compounds was previously used in reference [12]. The theoretical and experimental bond lengths and angles were compared using the correlation coefficient (R) and the mean absolute error (MAE). The MAE is determined as the average value of the absolute difference between two data sets. When comparing the bond lengths of the **Eu-PLSC** complex, the correlation coefficient was 0.99, while the MAE value was 0.03 Å (Table S1). The experimental bond lengths with phenyl oxygen atoms were 2.224 and 2.282 Å, while the theoretical values were 2.226 and 2.308 Å. The bond lengths with carbonyl oxygen atoms displayed absolute differences of 0.070 and 0.017 Å, while the average difference for the interactions with azomethine nitrogen atoms was 0.117 Å. The bond lengths were well-reproduced, as the bonds with the protonated PLSC ligand were longer than those with the deprotonated ligand, which coincides with the experimental data. The differences in bond lengths are partially due to the system’s relaxation, as the optimization was performed for the isolated molecule in a vacuum, without counterions and water molecules. However, it should be emphasized that two PLSC ligands and Eu(III) represent the rigid part of the examined molecule, whereas aqua and nitrato ligands are more flexible. The experimental and theoretical bond lengths between the central metal ion and aqua ligand are 2.432 and 2.537 Å. The bond lengths for Eu−O from nitrato ligands are 2.578/2.626 Å in the crystallographic structure and 2.477/2.447 Å in the optimized structure. These values differ slightly from those previously examined. The bond lengths within protonated and deprotonated PLSC ligands were much better reproduced, with the MAE value being only 0.009 Å, consistent with the experimental value. This result is expected as the PLSC ligand is characterized by extensive delocalization between the phenyl ring, two nitrogen atoms, carbonyl, and amino groups, which restricts free vibrations of the included groups.

Due to the complexity of the **Eu-PLSC** structure, a large number of experimental and theoretical bond angles are listed, as shown in Table S2. Overall, good reproducibility of the experimental values was found, with a correlation coefficient of 0.98 and an MAE value of 3.4°, although certain differences were present. The experimental and theoretical angles formed between phenyl oxygens and the central metal ion were 88.5 and 93.3°, respectively. A similar difference was observed for the angle between carbonyl oxygens and Eu(III), which was 140.6 vs. 143.6°. When considering the angles between phenyl oxygen, europium(III), and carbonyl oxygen, the experimental/theoretical values of the protonated (132.2/126.5°) and deprotonated PLSC ligands (131.7/130.1°) were also well-reproduced. A slight change in the position of the central metal ion in the theoretical structure occurred as a result of energy minimization. Much larger differences were found in the angles involving aqua and nitrato ligands. The absolute difference for the angle O4−Eu−O8 was 11.7°, while for O4−Eu−O7 it was 12.6°. As previously explained, the position of the nitrato ligand is dependent on interactions with the neighboring units, and in the optimized structure, this ligand is slightly shifted. This is also reflected in the difference in angle denoted as Eu−N9−O9 (18.6°). The angles formed by the atoms of the PLSC ligand were much more similar between the experimental and theoretical structures. The MAE value calculated using only these angles was 0.9°, which supports the assumption that the rigidity of the ligand is the primary reason for the obtained result. Nevertheless, it can be concluded that the optimized structure represents the experimental one well and can be used for further analysis of the interactions and comparison with **Fe-PLSC**.

The structure of **Fe-PLSC** was optimized at the B3LYP/6-311++G(d,p)(H,C,N,O,Cl)/def2-TZVP(Fe) level of theory, together with the chloride anion. The optimized and experimental bond lengths are presented in Table S4. The correlation coefficient and MAE values for bond angles were 0.99 and 0.029 Å. The structure of the complex is significantly simpler, and its structural parameters are less dependent on the other species present in the experimental structure. The theoretical bond lengths, including those of the phenyl oxygen, azomethine nitrogen, carbonyl oxygen, and Fe(III), are 1.914, 1.978, and 1.943 Å, respectively. These values in the optimized structure are lower than those in **Eu-PLSC**, as experimentally observed. The experimental and theoretical bond lengths, denoted as Fe−O4 (water molecule), are 2.109 and 2.083 Å, respectively, which indicates that the **Fe-PLSC** structure is less prone to changes upon optimization. The absolute differences in values of bond lengths between chlorido ligands and the central metal ion are 0.020 and 0.051 Å. Again, the theoretical bond lengths within the protonated PLSC ligand were almost identical to the experimental ones, with the absolute differences ranging from 0.003 to 0.033 Å.

The correlation coefficient and MAE values for the bond angles of **Fe-PLSC**, when compared, are 0.98 and 2.77° (Table S5). The most notable difference of 16.2° was found between phenyl oxygen, Fe(III), and carbonyl oxygen atoms. The pseudo-octahedral geometry is distorted by the change in position of the chlorido ligands, with differences of around 10°. These atoms interact with neighboring **Fe-PLSC** molecules, as shown in the previous section, resulting in a slight change in the angle value upon optimization. The remaining angles differ by less than 3°. The stability of the PLSC ligand’s structure is proven for this complex as well. These differences are explained in the section on QTAIM analysis of the interactions. Based on these results, it can be stated that the theoretical **Fe-PLSC** structure also exhibits a high resemblance to the experimental one.

### 2.5. QTAIM Analysis of Interactions

The interactions between central metal ions and donor atoms are examined using QTAIM analysis, as this methodology is suitable for describing the coordination environment and calculating the metal–ligand interaction energies in f-element compounds [40,41,42]. Several parameters of the bond critical points (BCPs) were calculated for the selected interactions: the electron density (*ρ*(r)), Laplacian (∇^2^*ρ*(r)), Lagrangian kinetic electron density (*G*(r)), potential electron density (*V*(r)), density of total electron energy (*H*(r) =*G*(r) + *V*(r)), and interatomic bond energy (E_bond_ = *V*(r)/2) [43]. Bader and Essen divided interactions into two groups based on the values of electron density at the bond critical points (BCPs). The first group consists of shared interactions (covalent bonds) with an electron density higher than 0.1 a.u. The second group comprises hydrogen bonds, ionic bonds, and van der Waals interactions, characterized by an electron density of approximately 0.01 a.u. [44,45]. A detailed classification was presented by Bianchi and coworkers, with the main criterion being the relative ratio of *G*(r) and *V*(r). The covalent interactions are characterized by a −*G*(r)/*V*(r) value lower than 1, the intermediate (transit) region covers interactions with partial covalent character and values between 1 and 2, and ionic bonds have a value of this parameter higher than 2 [46]. Additionally, the examination of the interaction type can be made when the total energy of electrons is examined [43]. Interactions with the negative value of this parameter can be considered covalent. The interatomic interaction energy was calculated from the potential electron density, as suggested by Espinosa [47]. Wu and coworkers demonstrated that the trends in QTAIM parameters were independent of the level of theory used for optimizing lanthanide complexes [48]. These parameters are listed in Table S7 for both compounds, and the BCPs are presented in Figure S9.

The QTAIM parameters were used to compare the characteristics of Eu−O interactions in **Eu-PLSC**. The highest value of electron density is found for the interaction between Eu(III) and the phenyl oxygen atom of the protonated PLSC ligand (0.073 a.u.). This interaction exhibits a significant covalent character, with a ratio of −*G*(r)/*V*(r) equal to 0.9 and *H*(r) being lower than 0 (−12.8 kJ mol^−1^) (Table S7). The interatomic bond energy has the lowest value of −120.6 kJ mol^−1^. When the same interaction with the deprotonated PLSC ligand is concerned, the electron density is 0.058 a.u., and there is a slightly positive *H*(r) value (0.4 kJ mol^−1^). The calculated bond energy is −85.2 kJ mol^−1^. As previously mentioned, delocalization within the phenyl ring enhances the interaction strength with the central metal ion. The carbonyl oxygen atoms (O5 and O2) interact with Eu(III) through partial covalent interactions. The electron densities are 0.045 (O5) and 0.039 a.u. (O2). These electron densities are well-reflected in the interaction energies, with the interaction Eu−O5 being stronger than Eu−O2 at −58.5 vs. −49.2 kJ mol^−1^. Again, the interactions with donor atoms of the protonated PLSC ligand are stronger than those with atoms of the deprotonated PLSC. Two oxygen atoms of the nitrato ligand show similar strength (−62.9 and −56.9 kJ mol^−1^), with a −*G*(r)/*V*(r) value of 1 and *H*(r) < 0, which proves their partial covalent character. The weakest interaction of the type Eu−O is the one with a water molecule (−44.0 kJ mol^−1^), as verified by the lowest electron density (0.037 a.u.) and positive *H*(r) value. Due to the position of the ligands, the interactions with azomethine nitrogen atoms show much lower electron densities (0.023 and 0.031 a.u.). These interactions have positive *H*(r) values—5.1 and 3.2 kJ mol^−1^—and interaction energies of −22.7 and −32.4 kJ mol^−1^ (Table S7). These results prove the assumption that the interaction strength can be modulated by changing the protonation/deprotonation of the PLSC ligand.

The overall stability of the complex is also influenced by the presence of several weak interactions. Some of these interactions are formed within structures of separate ligands, such as the weak interaction between the methylhydroxy oxygen atom and a pyridine hydrogen, with an interaction energy of −18.0 kJ mol^−1^ and an electron density of 0.017 a.u. Azomethine nitrogen interacts with the phenyl oxygen atom (O1···N2) through a weak interaction (Table S7). There is also an interaction between the atoms of two ligands, such as the one between the nitrogen atom of the aliphatic chain in the protonated PLSC ligand and the methyl group of the deprotonated PLSC ligand. This type of interaction is very weak, with an interaction energy of −1.8 kJ mol^−1^ and a −*G*(r)/*V*(r) value equal to 1.4. During the optimization of the structure, stabilization occurred due to the interaction between the aqua and nitrato ligands, which influences the change in the angles enclosed by atoms belonging to these species, as discussed in the previous section. This relatively strong interaction has an energy of −19.7 kJ mol^−1^ and a positive *H*(r) value.

When interactions within **Fe-PLSC** are examined, the strongest Fe−O bond is formed between the central metal ion and carbonyl oxygen atom (−199.1 kJ mol^−1^). The electron density is much higher than those discussed for Eu−O, 0.091 a.u., with a negative value of *H*(r) (−21.5 kJ mol^−1^) and a value of −*G*(r)/*V*(r) lower than 1. A high electron density value is also present between Fe(III) and the phenyl oxygen atom (0.077 a.u.) with an interaction energy of −164.6 kJ mol^−1^. In the structure of **Fe-PLSC**, the weakest interaction when metal–ligand interactions are examined is between the water molecule and Fe(III). The interaction strength is −113.8 kJ mol^−1^, although the partial covalent character is determined through a negative value of the total energy of the electron density (Table S7). The interaction between azomethine nitrogen and iron(III) is much stronger than in the case of **Eu-PLSC**. This is, in fact, the strongest interaction within the **Fe-PLSC** structure, with an energy of −214.8 kJ mol^−1^. Two chlorido ligands also interact similarly with Fe(III). These interactions have energies of −122.0 and −120.4 kJ mol^−1^ and a partial covalent character (−*G*(r)/*V*(r) = 0.8). As the structure of the neutral **Fe-PLSC** complex, with a chloride counterion present, was subjected to QTAIM analysis, there are additional stabilization interactions. One is formed between the aqua ligand and the chloride ion (HOH···Cl3), with an interaction energy of −23.2 kJ mol^−1^. The low distance between the protonated nitrogen atom of the aliphatic chain and the chloride anion is also responsible for forming the interaction that is denoted as N2H···Cl3. This interaction has an electron density of 0.054 a.u. and an energy of −58.6 kJ mol^−1^. In the solution, the existence of these interactions is not expected. Another weak interaction was identified between the oxygen atom of the methylhydroxyl group and pyridine hydrogen, with an energy of −10.8 kJ mol^−1^.

### 2.6. Experimental and Theoretical HSA-Binding Affinity

**Figure 7:**
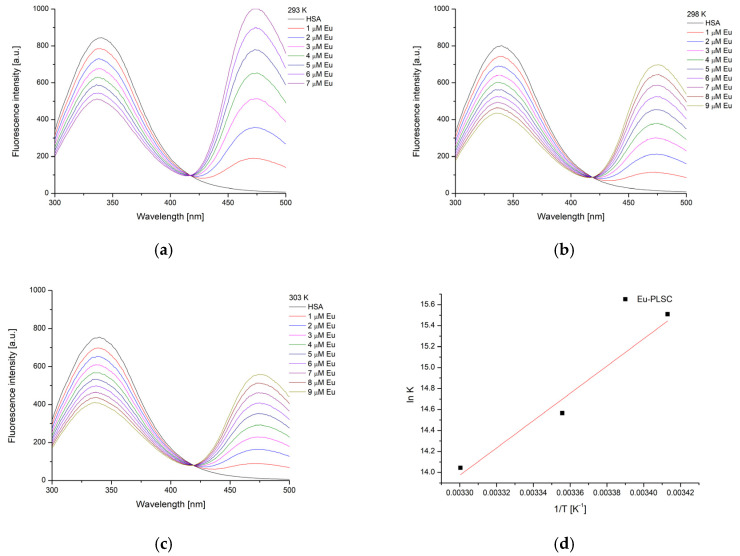
The fluorescence spectra of HSA after the addition of various concentrations of **Eu-PLSC** at (**a**) 20, (**b**) 25, and (**c**) 30 °C and (**d**) Van ’t Hoff diagram for the binding process of **Eu-PLSC** and HSA.

**Figure 8:**
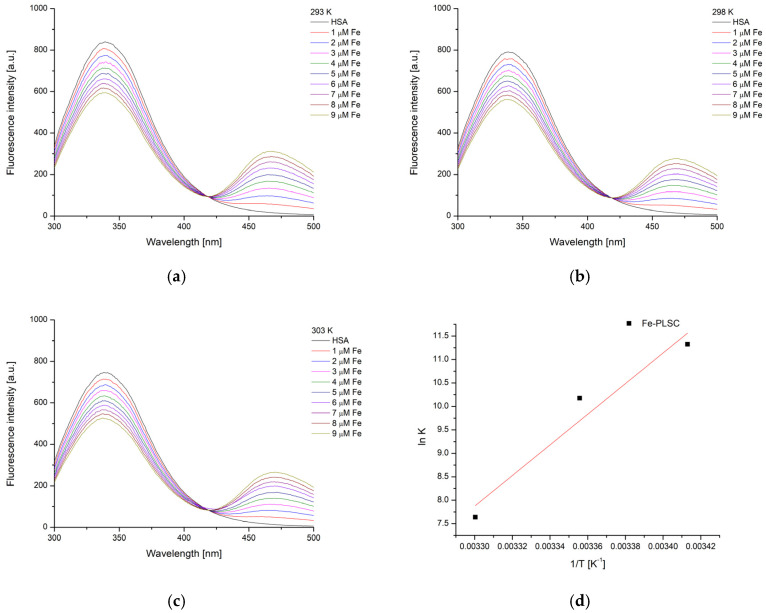
The fluorescence spectra of HSA after the addition of various concentrations of **Fe-PLSC** at (**a**) 20, (**b**) 25, (**c**) 30 °C and (**d**) Van ’t Hoff diagram for the binding process of **Fe-PLSC** and HSA.

Human serum albumin (HSA) is the most abundant protein in human blood plasma, playing a crucial role in maintaining osmotic pressure and transporting a wide range of endogenous and exogenous substances, including drugs, hormones, and fatty acids [49]. Due to its strong binding capacity and well-defined structure, HSA is frequently used as a model protein in biochemical and pharmacological studies. Its intrinsic fluorescence, primarily from a single tryptophan residue (Trp214), makes HSA a valuable tool in fluorescence spectroscopy for investigating ligand-binding interactions, conformational changes, and protein dynamics. The binding activity towards HSA was investigated by spectrofluorimetric titration to examine the binding of europium(III) and iron(III) complexes to the protein’s active site. The HSA solution was irradiated by the excitation wavelength of 280 nm, which is sufficient for the excitation of tryptophan and tyrosine residues [50]. The electronic emission spectra of HSA, with varying concentrations of the obtained complexes, are presented in Figure 7 and Figure 8. The fluorescence emission intensity of the HSA solution was corrected according to Equation (1), as both complexes absorbed at 280 nm, as presented in Figures S10 and S11.

Upon the addition of the complexes, the intensity of the HSA fluorescence emission significantly decreased, although it should be noted that this decrease was much faster in the case of **Eu-PLSC**. In both cases, a new band emerged with a maximum at 475 nm (**Eu-PLSC**) and 468 nm (**Fe-PLSC**), signifying the formation of a new fluorescent species. This result is consistent with previous studies on the interaction between europium(III) complexes and HSA [51]. The isosbestic point was present at 419 and 418 nm, respectively. The formation of the second peak was attributed to the fluorescence of the complexes, which underscored the need for correcting the intensity of the HSA fluorescence emission, as explained in the Section 3. The binding constants were between 5.44 × 10^6^ and 1.26 × 10^6^ M^−1^ for **Eu-PLSC** in the experimental temperature range. The decrease in the stability of complexes with increasing temperature is likely due to molecular motion within the formed complexes. The binding of the **Eu-PLSC** complex is stronger compared to the Eu(III) complex containing a lawsone ligand (K_b_ = 3.25 × 10^5^ M^−1^), which was described as moderate [22]. In the paper by Song and coworkers, a bimetallic europium complex with hydrazine Schiff base showed lower binding constants between 0.28 and 1.66 × 10^4^ M^−1^ [51]. When interactions between **Fe-PLSC** and HSA were examined, the binding constants were found to be lower by two orders of magnitude, ranging from 8.29 × 10^4^ to 2.08 × 10^3^ M^−1^. The binding constants of the Fe(III) complex containing 2-((1H-tetrazol-1-yl)methylene)-1H-imidazole-4,5-dicarboxylic acid were comparable to the values from this contribution, decreasing in value with an increase in temperature [50]. From a structural analysis of complexes, it can be expected that the presence of protonated and deprotonated PLSC, nitrato, and aqua ligands plays an essential role in the stability of the formed interactions. The change in binding constants with temperature allowed for the calculation of the thermodynamic parameters of binding.

The change in binding enthalpy for the obtained complexes is negative, suggesting that the energy process is exothermic, and its value was calculated to be −108.3 kJ mol^−1^ for **Eu-PLSC** and −271.4 kJ mol^−1^ for **Fe-PLSC** (Table 1). This means that the binding of **Eu-PLSC** is stronger in terms of the enthalpic contribution to the binding energy. The change in binding entropy for both complexes was negative; for **Eu-PLSC**, it was −241.1 J mol^−1^ K^−1^, and for **Fe-PLSC**, it was −830.2 J mol^−1^ K^−1^. This led to the conclusion that between the HSA and these complexes, specific interactions such as hydrogen bonds and strong electrostatic interactions lead to the ordering of the system. The change in Gibbs free energy of binding was between −37.6 and −35.2 kJ mol^−1^ for **Eu-PLSC** and between −28.2 and −19.9 kJ mol^−1^ for **Fe-PLSC**, which proved the spontaneity of the binding process for both complexes. Considering these values, it can be concluded that **Eu-PLSC** binds strongly, and the formed complex is more stable than **Fe-PLSC**. High ∆G_b_ values indicate that since the **Eu-PLSC** is nine-coordinated, it is capable of realizing a greater number of interactions of different types, especially hydrogen bonds and Coulomb electrostatic interactions that give a significant contribution to the total interaction energy. The values of the binding constants decrease with increasing temperature, indicating that the quenching mechanism of HSA fluorescence is of a static type in this case. Compared to ibuprofen, whose binding energy to the Sudlow site (DS2) is −18.0 kJ mol^−1^, these two complexes are good candidates for further studies of transport through the blood to sites of potential anticancer activity.

**Figure 9:**
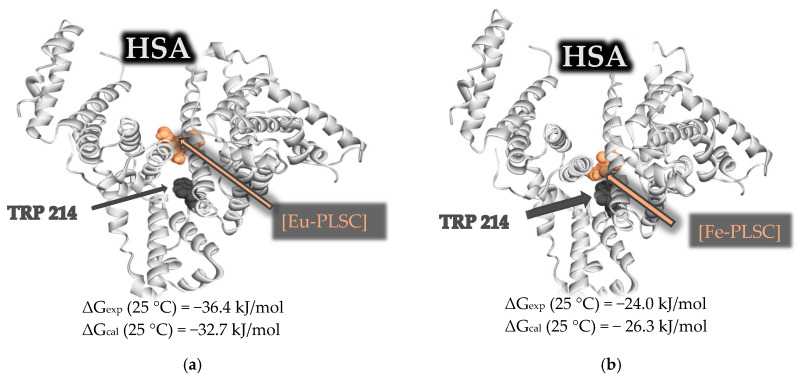
HSA molecule (PDB ID: 6EZQ) with bound ligands, (**a**) **Eu-PLSC** complex and (**b**) **Fe-PLSC** complex, occupying the FA7 binding site. Ligands and tryptophan are depicted using ball representation. Eu(III) and Fe(III) complex are shown in light orange, and Trp214 is represented in dark grey.

**Figure 10:**
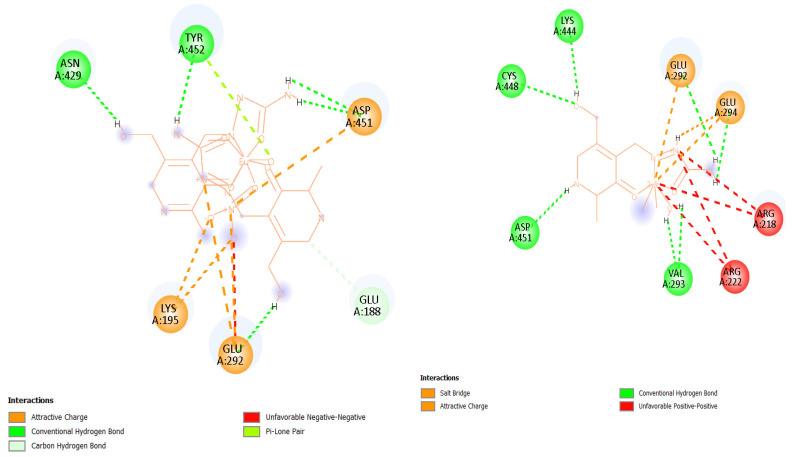
Interactions in the FA7 binding site between amino acids and **Eu-PLSC** (**left**) and **Fe-PLSC** (**right**).

Molecular docking simulations were performed to investigate the binding interactions between human serum albumin (HSA, PDB ID: 6EZQ) and two metal complexes: a nine-coordinated **Eu-PLSC** and a six-coordinated **Fe-PLSC**. Previously obtained changes in Gibbs free energy of binding were −36.4 (**Eu-PLSC**) and −24.0 kJ mol^−1^ (**Fe-PLSC**) at 298 K. The best binding configuration for both complexes was at the FA7 binding site, depicted in Figure 9. **Eu-PLSC** had a change in Gibbs free energy of −32.7 kJ mol^−1^, while **Fe-PLSC** had −26.3 kJ mol^−1^, comparable to the experimentally obtained values. Analysis of the docking poses showed that the europium(III) complex formed multiple stabilizing interactions within the FA7 binding site of HSA, including hydrogen bonds (classic and carbon) with ASN429, TYR452, and GLU292; electrostatic interactions (unfavorable negative-negative and attractive charge) with LYS195, GLU292, and ASP451; and π-LP contacts with TYR452 (Figure 10). Conversely, the iron(III) complex formed hydrogen bonds (classic, carbon, and salt bridges) with LYS444, CYS448, ASP451, and VAL293 as well as electrostatic interactions with GLU292 and GLU294. There were also two unfavorable interactions with ARG218 and ARG222 (Figure 10). These interactions were primarily located near Sudlow’s site I, suggesting a high-affinity binding mode. In contrast, while still capable of binding to HSA, the iron complex demonstrated fewer and weaker interactions, mostly involving polar contacts and less extensive hydrophobic stabilization. The higher coordination number and larger ionic radius of the europium(III) ion may contribute to its enhanced interaction capability, allowing it to engage with more residues within the binding site. The **Eu-PLSC** displayed superior binding characteristics toward HSA, reflected by its more negative Gibbs free energy and denser interaction network.

### 2.7. Experimental and Theoretical DNA-Binding Affinity

Deoxyribonucleic acid (DNA) is the central genetic material in all living organisms, carrying the instructions for cellular structure, function, and replication. Due to its critical role in cell proliferation and survival, DNA is a primary target in the development of anticancer drugs. Many chemotherapeutic agents exert their activity by binding to DNA, disrupting its replication and transcription, ultimately leading to cell cycle arrest or apoptosis. DNA-binding interactions can occur through intercalation, groove binding, or covalent attachment, and understanding these interactions is essential for evaluating the biological potential of metal-based compounds [52]. Furthermore, DNA exhibits intrinsic fluorescence, although weak, which can be significantly altered upon binding with metal complexes or fluorescent ligands. This change in fluorescence is widely exploited in spectroscopic studies to probe the mode and strength of DNA binding, making it a valuable tool in the design and screening of new anticancer agents.

**Figure 11:**
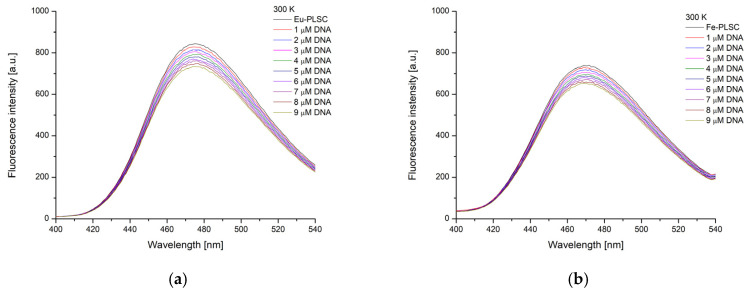
The fluorescence spectra of (**a**) **Eu-PLSC** and (**b**) **Fe-PLSC** without and with various concentrations of CT-DNA at 27 °C.

Both **Eu-PLSC** and **Fe-PLSC** showed stable fluorescence upon irradiation at 280 nm (Figures S10 and S11). The fluorescence emission spectra were recorded between 300 and 550 nm (Figure 11) upon the addition of CT-DNA, as explained in reference [53]. The excitation and emission slits for **Eu-PLSC** were set to 10 nm and 5 nm, respectively, and for **Fe-PLSC**, both slits were set to 10 nm due to the low fluorescence emission. The emission maxima are located at 475 nm (**Eu-PLSC**) and 470 nm (**Fe-PLSC**). The fluorescence spectra were recorded at 27 °C. The K_SV_ values, binding constants, number of binding positions (Hill coefficient), and change in Gibbs free energy of binding are presented in Table 2.

Based on the experimental results obtained for the fluorescence quenching of europium(III) and iron(III) complexes upon binding to CT-DNA, it is concluded that the binding process is spontaneous. Comparison of the absolute values of the Gibbs free energies of binding led to the conclusion that the **Fe-PLSC** complex binds more strongly to CT-DNA than the **Eu-PLSC** complex, which was to be expected given that the **Eu-PLSC** complex is extremely voluminous relative to **Fe-PLSC** for intercalation. Compared to the nickel complex experimentally studied in reference [33], these two complexes showed significantly stronger binding to the CT-DNA molecule at 27 °C, making them candidates for further research in the field of blocking tumor DNA replication. These experimental results are further examined through molecular docking simulations to verify the mode of interaction with DNA that was obtained.

#### 2.7.1. Ethidium Bromide Displacement Studies

Ethidium bromide (EB) displacement studies examine the binding mechanism of different compounds to DNA [54]. This method is based on replacing EB and comparing the interaction strength. The intercalation of EB in the DNA structure, as a result, results in the formation of a highly fluorescent species CT-DNA-EB with an excitation wavelength of 520 nm and emission maximum at 600 nm [55]. Upon the addition of the complex, the structure of CT-DNA-EB is disrupted, as observed by the decrease in fluorescence emission intensity. The unbound EB is easily quenched by the present species in the solution.

**Figure 12:**
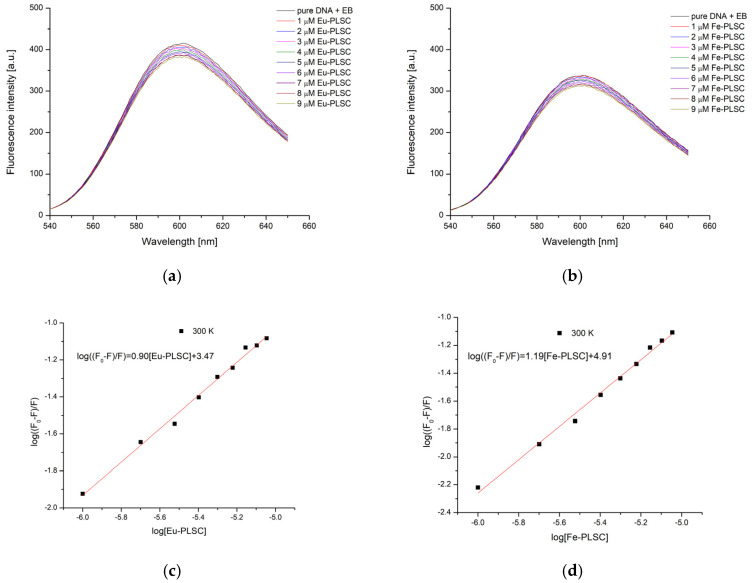
Fluorescence emission spectra of CT-DNA for the titration with the (**a**) **Eu-PLSC** and (**b**) **Fe-PLSC**; the double-log Stern–Volmer dependency of intensity on the concentration of (**c**) **Eu-PLSC** and (**d**) **Fe-PLSC**.

The emission spectra of CT-DNA-EB in the presence of various concentrations of the complexes at 27 °C are shown in Figure 12. As expected, the emission maxima decrease as the concentration of the obtained complexes increases. The Stern–Volmer constant for this process for the **Eu-PLSC** complex is 9.69 × 10^3^ M^−1^ and for **Fe-PLSC** is 8.17 × 10^3^ M^−1^. The previously investigated nickel(II) complex with the pyridoxal-semicarbazone (PLSC) ligand showed K_SV_ values that were an order of magnitude lower than the one obtained in this experiment, which is a result of ligand stability through delocalization of the structure [33]. The binding constants obtained through the double log SV equation for **Eu-PLSC** and **Fe-PLSC** are 2.93 × 10^3^ and 8.24 × 10^4^ M^−1^, respectively. The value of n equals 0.90 for **Eu-PLSC** and 1.19 for **Fe-PLSC** (Figure 12). These values suggest that one molecule of ethidium bromide is displaced per complex molecule; however, the mechanism of displacement may involve either intercalation or groove binding, depending on the size and geometry of the complex, as determined in the molecular docking section. The binding affinity, calculated from the binding constant, is −19.9 kJ mol^−1^ for **Eu-PLSC** and −28.2 kJ mol^−1^ for **Eu-PLSC**.

#### 2.7.2. Molecular Docking Simulations of the Binding Mechanism to DNA

**Figure 13:**
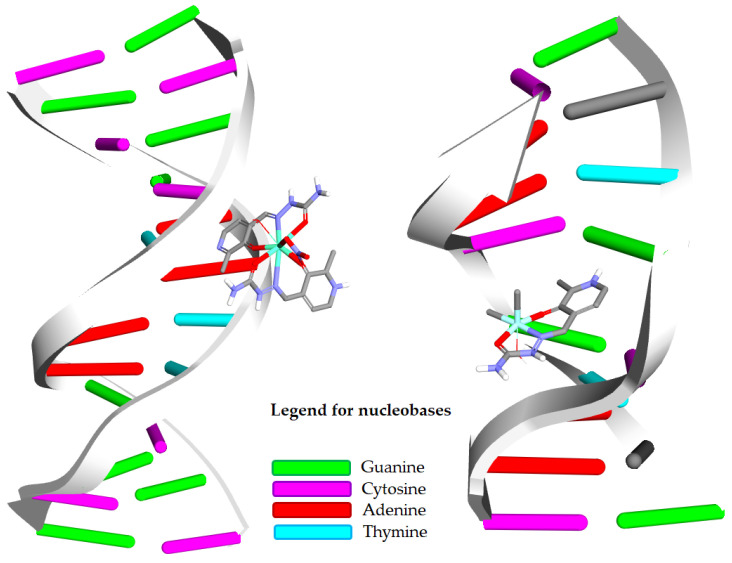
DNA molecule PDB ID: 1BNA (**left**) and 454D (**right**) with bound ligands: (**left**) **Eu-PLSC** complex and (**right**) **Fe-PLSC** complex.

**Figure 14:**
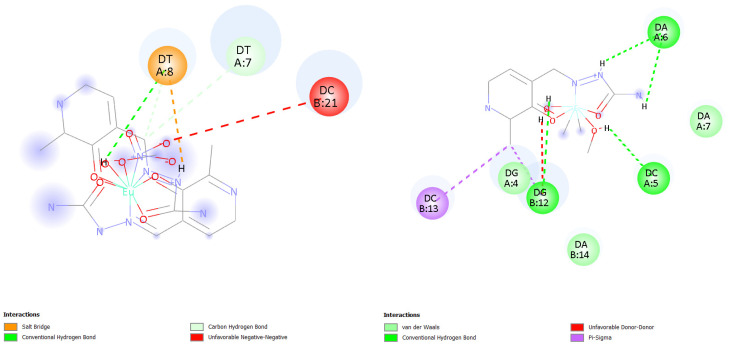
Interactions with DNA in binding sites between nucleobases and **Eu-PLSC** (**left**) and **Fe-PLSC** (**right**).

Molecular docking studies were carried out to evaluate the binding interactions between CT-DNA and europium(III) and iron(III) complexes. The results demonstrated a clear difference in binding behavior, with the **Eu-PLSC** complex showing a significantly stronger affinity for minor groove binding to DNA (PDB ID: 1BNA). The europium(III) complex was found to interact with the 1BNA DNA structure, predominantly through minor groove binding (Figure 13). It formed a rich network of interactions, including salt bridge hydrogen bonding with phosphate groups and a sugar ring, carbon–hydrogen bonding, and an electrostatic repulsion interaction between nitrate and the phosphate group (Figure 14). The experimentally determined value of the change in Gibbs free energy of binding was −21.7 kJ mol^−1^, while the calculated value is also −21.7 kJ mol^−1^, representing an excellent agreement. In contrast, **Fe-PLSC** preferred an intercalation interaction with DNA (PDB ID: 454D) (Figure 13). Iron(III) complex created different types of interactions compared to **Eu-PLSC**, such as van der Waals dispersion interactions, hydrogen bonding, unfavorable donor–donor interaction, and π–σ interactions with nucleobases, as shown in Figure 14. In this case, the experimentally obtained (−26.2 kJ mol^−1^) and calculated values (−26.5 kJ mol^−1^) of change in Gibbs free energy of binding are also in good agreement. **Fe-PLSC** interacted with the entire spatial bulk in the intercalation site with nucleobases, displaying more hydrogen bonds, van der Waals, and π–σ interactions. Details for the interactions with DNA are given in Table S8. These factors collectively contribute to the superior docking score and thermodynamic favorability observed for the iron(III) complex. Considering that the **Eu-PLSC** complex is nine-coordinated and voluminous, it is not expected to be capable of intercalation. At the same time, **Fe-PLSC** is more suitable for this type of interaction.

## 3. Materials and Methods

### 3.1. Chemicals

All chemicals and solvents used for synthesis and protein/DNA-binding affinity analysis were obtained from Merck (Darmstadt, Germany) and used without further purification. The PLSC ligand was prepared as explained in the reference [36].

### 3.2. Synthesis of [Fe(PLSC)Cl_2_(H_2_O)][Cl] (**Fe-PLSC**)

Twenty milliliters of water were added to an Erlenmeyer flask containing a PLSC ligand mixture (0.1 mmol) and an equal amount of FeCl_3_·6H_2_O. Heating the mixture resulted in complete dissolution, yielding a dark brown, clear solution. After a week, dark brown single crystals of the Fe complex were isolated. Yield: 0.25 g (77%). Anal. Calcd. for [Fe(PLSC)Cl_2_(H_2_O)][Cl] (C_9_H_14_FeN_4_O_4_, 415.03): C, 26.73; H, 3.49; N, 13.86. Found: C, 26.68; H, 3.60; N, 13.73.

### 3.3. Synthesis of [Eu(PLSC)(PLSC-H)(NO_3_)(H_2_O)][NO_3_]·3(H_2_O) (**Eu-PLSC**)

Ligand PLSC (0.1 mmol) was dissolved in 15 mL of water and gradually heated to a boil. An equivalent amount (0.1 mmol) of Eu(NO_3_)_2_·3H_2_O salt was added to the hot ligand solution. With the addition of Eu salt, sudden turbidity occurred, after which the solution was filtered, leaving a clear yellow solution to crystallize at room temperature. For nearly a month, the solution was observed daily; initially, it became cloudy every few days, prompting filtration. Subsequently, turbidity diminished, and the clear solution crystallized into yellow-orange single crystals. Yield: 0.19 g (58.6%). Anal. Calcd. for [Eu(PLSC)(PLSC-H)(NO_3_)(H_2_O)][NO_3_]·3(H_2_O) (C_18_H_31_EuN_10_O_16_, 795.49): C, 27.18; H, 3.93; N, 17.61. Found: C, 27.09; H, 3.98; N, 17.59.

### 3.4. Spectral Characterization

The vibrational spectra of the **Eu-PLSC** and **Fe-PLSC** complexes were recorded on a Thermo Nicolet-Avatar 370 FTIR spectrometer (Thermo Fisher Scientific, Waltham, MA, USA) in the range 4000–600 cm^−1^, with a resolution of 0.5 cm^−1^. For this purpose, 2 mg of complex was mixed with 150 mg of potassium bromide, and a pellet was prepared. The UV–VIS spectra were obtained on a Thermo Scientific UV–VIS Spectrometer (Thermo Fisher Scientific, Waltham, MA, USA) between 800 and 200 nm. The resolution was set to 1 nm with an integration time of 0.20 s. Experimental spectra were visualized and processed in the Origin program 2018 (9.5) [56]. The elemental analysis of air-dried samples was performed on an Elementar Vario El III (Elementar Analysesysteme GmbH, Langenselbold, Germany). The mass percentages were calculated based on the intensity corresponding to different oxides.

### 3.5. X-Ray Crystallographic Analysis

A yellow prismatic crystal of **Eu-PLSC**, measuring 0.193 × 0.139 × 0.048 mm, was selected and mounted on a nylon cryoloop. Diffraction data were collected at 123 K using MoKα radiation (λ = 0.71073 Å) on a Rigaku Synergy S diffractometer equipped with a HYPIX 6000 hybrid photon counting detector. For **Fe-PLSC**, a small red-orange plate crystal with dimensions of 0.040 × 0.037 × 0.018 mm was selected and examined using CuKα radiation (λ = 1.54184 Å). Data were collected and processed, including an empirical (multi-scan) absorption correction, using the CrysAlisPro software (https://rigaku.com/products/crystallography/x-ray-diffraction/crysalispro, accessed on 7 May 2025) [1]. The structure was solved and refined through standard methods utilizing the SHELX software (https://shelx.uni-goettingen.de/, accessed on 7 May 2025) suite in conjunction with the Olex2 graphical interface [2,3]. Non-hydrogen atoms were refined with anisotropic displacement ellipsoids, while hydrogen atoms bonded to carbon were placed in calculated positions based on a riding model. The positions of hydrogen atoms bonded to oxygen and nitrogen were derived from the difference Fourier map and were refined without restraint.

The crystallographic structures of complexes were deposited in the Cambridge Crystallographic Data Centre (CCDC, 12 Union Road, Cambridge CB2 IEZ, UK; e-mail: depos-it@ccdc.cam.ac.uk) under the following CCDC numbers: 2,420,972 for [Eu(PLSC)(PLSC-H)(NO_3_)(H_2_O)][NO_3_] × 3(H_2_O) and 2,420,971 for [Fe(PLSC)Cl_2_(H_2_O)][Cl]. Additional details concerning these structures are provided in Table 3.

### 3.6. Hirshfeld Surface Analysis

The stability of crystal packaging greatly depends on the intramolecular interactions between compounds in unit cells. The comparison of these interactions in the crystal structures of **Eu-PLSC** and **Fe-PLSC** complexes was done through Hirshfeld surface analysis in the CrystalExplorer [57] program. The interactions within this approach were examined by preparing a graph characterized by two distances, one between two nearest nuclei (d_e_) and the other connecting nuclei with the external surface (d_i_) [58,59,60]. The points on the surface are colored depending on the value of the distance when compared to the van der Waals separations between atoms. If the distance is shorter, equal to, or longer than these separations, the color is red, white, or blue, respectively. The most numerous contacts between atoms are discussed in the main text, while fingerprint plots are shown in the Supplementary Information. The normalized distances are presented between −0.6180 (red) and 1.1825 (blue) for **Eu-PLSC** and between −0.5698 (red) and 1.1414 (blue) for **Fe-PLSC**.

### 3.7. Theoretical Structural Analysis

The experimental structures of the obtained complexes were used for the theoretical structural analysis in the Gaussian 09 program package (Gaussian 09, revision C 01) [61]. The B3LYP functional [62] was applied, with the basis set dependent on the size of the complex, 6-311++G(d,p)(H,C,N,O,Cl)/def2-TZVP(Fe) and 6-31+G(d,p)(H,C,N,O)/def2-TZVP(Eu) [63,64,65,66], as obtained from the Basis Set Exchange [67]. The optimizations were done without any geometrical constraints. The absence of imaginary frequencies was used to prove that the minimum of the potential energy surface was reached. The interactions between central metal ion and ligands were assessed through the Quantum Theory of Atoms in Molecules (QTAIM) approach, as proposed by Bader [68,69], in the AIMAll program package [70]. These calculations were performed on a .wfx file from the Gaussian 09 program optimization. Electron density, Laplacian, and other parameters were calculated for the bond critical points (BCP) [71]. Optimized structures were visualized in the GausView program [72].

### 3.8. Spectrofluorimetric Determination of HSA Binding Affinity

The protein binding affinity was determined through spectrofluorimetric titration on a Cary Eclipse MY2048CH03 spectrophotometer (Agilent Technologies, Santa Clara, CA, USA). The scan rate was 600 nm min^−1^, with the excitation and emission slits being 10 and 5 nm, respectively. The standard solution of HSA (5 × 10^−6^ M) was prepared in phosphate buffer saline at pH = 7.4 (concentrations of NaCl and KCl were 137 and 2.7 mM). The excitation wavelength was set to 280 nm, which activates the tryptophan residues in the protein structure. The emission spectra were recorded in the range between 300 and 500 nm. The concentration of complexes ranged between 1 and 9 µM. As both complexes absorbed at the excitation and emission wavelengths of the protein, the emission intensity was corrected using the following equation [73,74]:

(1) $$F_{c}=F_{m}e^{\frac{A_{1}+A_{2}}{2}}$$

In the previous equation, F_c_ and F_m_ are the corrected and measured fluorescence emission intensities, while A_1_ and A_2_ are the absorbances of complexes at 280 and 350 nm [73]. Only the corrected intensities are shown in the manuscript.

As the fluorescence intensity of protein was dependent on the concentration of metal complexes, the double-log Stern–Volmer equation was applied [75]. This equation calculates the dependency of emission intensity on concentration, where F_0_ and F are the fluorescence emission intensity of the HSA solution without and with complexes, K_b_ is the binding constant, and n is the number of binding positions:

(2) $$\log\left(\frac{F_{0}-F}{F}\right)=\log K_{b}+n\log\left[complex\right]$$

The thermodynamic parameters (change in enthalpy, entropy, and Gibbs free energy of binding) were determined from the dependency of the binding constant on temperature, as shown in the following equations:

(3) $$\ln K_{b}=-\frac{∆H_{b}}{RT}+\frac{∆S_{b}}{R}$$

(4) $$∆G_{b}=∆H_{b}-T∆S_{b}$$

### 3.9. Spectrofluorimetric Determination of DNA Binding Affinity

The binding assay to the DNA molecule was followed through complex fluorescence quenching by CT-DNA. For these purposes, calf thymus DNA was chosen. The concentration of CT-DNA was measured following the absorbance at 260 nm (molar absorption coefficient 6600 dm^3^ mol^−1^ cm). These experiments were conducted using a Thermo Scientific Evolution 220 spectrophotometer (Thermo Fisher Scientific, Waltham, MA, USA). The concentration of the complex was kept constant at 10^−5^ M. The excitation wavelength (280 nm) was selected from the absorption spectrum because it gave optimal and measurable fluorescence intensity. The electronic emission spectra were recorded between 300 and 600 nm, and the excitation slit and emission slit were set to be 10 nm and 5 nm for **Eu-PLSC** and 10 nm both for **Fe-PLSC**. The stock solution of CT-DNA (0.474 mM) in phosphate buffer saline was added stepwise, and fluorescence spectra were recorded two minutes after addition.

For these interactions, the Stern–Volmer equation was also applied:

(5) $$\frac{F_{0}}{F_{Q}}=1+k_{Q}t_{Q}\left[Q\right]=1+K_{SV}[Q]$$

where F_0_/F_Q_ represents the fluorescence intensity ratio in the absence/presence of the quencher molecule; K_SV_ is the Stern–Volmer quenching constant; k_Q_ is the quenching rate constant; and τ_0_ is fluorophore’s (metal complex) lifetime.

The spectrofluorimetric titration was also used for the ethidium bromide (EB) displacement studies in the presence of the obtained complex. The concentrations of CT-DNA and EB were held constant at 50 and 5 μΜ in phosphate buffer saline, pH = 4 (concentrations of NaCl and KCl were 137 and 2.7 mM). The concentration of the complex was changed from 1 to 9 μM in steps of 1 μM. The excitation wavelength was set to 520 nm, and the emission spectra were recorded between 540 and 650 nm. The excitation slit was set to 5 nm, and the emission slit was set to 10 nm. The thermodynamic parameters of the displacement were determined from the binding constant.

### 3.10. Molecular Docking

The AutoDock 4.2 software [76] was used to examine the binding affinity of the investigated compounds. The crystal structures of the examined receptors (PDB IDs: 6EZQ [77], 1BNA [78], and 454D [79]) were extracted from the RCSB Protein Data Bank in PDB format. The target receptors were prepared for docking by removing the co-crystallized ligand, water molecules, and cofactors. For this purpose, BIOVIA Discovery Studio 4.0 [80] was employed. The AutoDockTools (ADT) [76] graphical user interface was used to calculate the Kollman partial charges and to add all hydrogens. The ligands were prepared for docking as explained in Section 3.7. The protein–ligand docking was done using the Lamarckian Genetic Algorithm (LGA) method [81]. The grid centers had dimensions of −29.447 × 7.056 × 30.274 Å^3^ for **Eu-PLSC** and −29.401 × 7.037 × 30.274 Å^3^ for **Fe-PLSC.** The binding affinity of the title molecules was investigated and discussed. Visualization and analysis of interactions and binding were performed using BIOVIA Discovery Studio Visualizer [80].

## 4. Conclusions

In this study, europium(III) and iron(III) complexes with the pyridoxal-semicarbazone (PLSC) ligand were synthesized and structurally characterized. The europium complex crystallized in a nine-coordinate geometry with both protonated and deprotonated forms of PLSC, while the iron complex adopted a six-coordinate environment with a single protonated PLSC ligand, two chlorido ligands, and one aqua ligand. The experimental structures were optimized using DFT methods, and the goodness of the applied models was validated by comparison with crystallographic data. Bond lengths and angles were closely reproduced for both complexes, with correlation coefficients of 0.99 and mean absolute errors (MAEs) of 0.03 Å for **Eu-PLSC** and 0.029 Å for **Fe-PLSC**. Bond angle MAE values were 3.4° and 2.77°, respectively, confirming the suitability of the applied computational approach.

QTAIM analysis supported the existence of partially covalent metal–ligand interactions. The highest electron density at bond critical points (BCPs) was found for Fe–O and Fe–N interactions, with associated interaction energies reaching up to −214.8 kJ mol^−1^. Weaker interactions, such as Eu–O(H_2_O) and intramolecular hydrogen bonds, were also identified and quantified, contributing to overall complex stability.

Biological relevance was demonstrated through binding studies with human serum albumin (HSA) and calf thymus DNA (CT-DNA). **Eu-PLSC** exhibited stronger binding to HSA, with Gibbs free energy of binding (ΔG) ranging from −37.6 to −35.2 kJ mol^−1^ and a maximum binding constant (K_b_) of 5.44 × 10^6^ M^−1^. In contrast, **Fe-PLSC** showed superior binding to CT-DNA, with ΔG = −26.2 kJ mol^−1^ and K_b_ up to 3.60 × 10^4^ M^−1^. Molecular docking confirmed these trends, indicating that **Eu-PLSC** binds at the FA7 site of HSA via hydrogen bonding and electrostatic interactions, whereas **Fe-PLSC** intercalates between DNA base pairs, stabilized by van der Waals and π-stacking interactions.

These results demonstrate that the coordination environment and protonation state of the PLSC ligand critically influence molecular geometry and biomolecular affinity. The excellent agreement between experimental and theoretical data and favorable thermodynamic parameters suggests that metal–PLSC complexes are promising candidates for further exploration in protein transport and DNA-targeted therapeutics.

## Figures and Tables

**Table 1:** Binding constant, ∆H_b_, ∆S_b_, ∆G_b,_ and the number of binding sites for HSA fluorescence quenching by **Eu-PLSC** and **Fe-PLSC**.

Complex	T [K]	K_b_ [M^−1^]	∆H_b_ [kJ mol^−1^]	∆S_b_ [J mol^−1^ K^−1^]	∆G_b_ [kJ mol^−1^]	n	R^2^
**Eu-PLSC**–HSA	293	5.44 × 10^6^	−108.3	−241.1	−37.6	1.39	0.994
	298	2.12 × 10^6^			−36.4	1.31	0.995
	303	1.26 × 10^6^			−35.2	1.27	0.998
**Fe-PLSC**–HSA	293	8.29 × 10^4^	−271.4	−830.2	−28.2	1.08	0.998
	298	2.62 × 10^4^			−24.0	0.99	0.998
	303	2.08 × 10^3^			−19.9	0.76	0.991

**Table 2:** Stern–Volmer quenching constant (K_SV_), binding constant (K_b_), ∆G_b_, and the Hill coefficient (n) for **Eu-PLSC** and **Fe-PLSC** binding to CT-DNA.

Complex	T [K]	K_SV_ [M^−1^]	K_b_ [M^−1^]	∆G_b_ [kJ mol^−1^]	n	R^2^
**Eu-PLSC**–CT-DNA	300	16.22 × 10^3^	6.05 × 10^3^	−21.72	0.92	0.997
**Fe-PLSC**–CT-DNA		14.55 × 10^3^	3.60 × 10^4^	−26.17	1.07	0.994

**Table 3:** Crystal data of the newly obtained complex.

Empirical Formula	[Eu(PLSC)(PLSC-H)(NO_3_)(H_2_O)][NO_3_] × 3(H_2_O) C_18_H_31_EuN_10_O_16_	[Fe(PLSC)Cl_2_(H_2_O)][Cl] C_9_H_14_FeN_4_O_4_
Formula weight	795.49	404.44
Temperature (K)	123(2)	123(2)
Crystal system	Triclinic	Orthorhombic
Space group	*P_−1_*	*P2_1_2_1_2_1_*
Radiation/wavelength (Å)	MoKα/0.71073 Å	CuKα/1.54184 Å
Volume (Å^3^)	1414.16(3)	1514.67(4)
Unit cell dimension (Å/°)	*a* = 9.03060(10) *b* = 11.97860(10) *c* = 13.8857(2) *α* = 73.8600(10) *β* = 78.7810(10) *β* = 80.0940(10)	*a* = 7.07760(10) *b* = 8.40230(10) *c* = 25.4703(2) *α* = 90(10) *β* = 90(10) *β* = 90(10)
Z	2	4
Volume (Å^3^)	1442.02(3)	1514.67(4)
Calculated density (g cm^−3^)	1.881	1.774
Goodness-of-fit on *F*^2^	1.040	1.078
h, k, l_max_	13, 17, 20	8, 7, 32
Data completeness	0.998	0.999
Θ range (°)	3.421–32.329	5.544–80.179
R1 [*I*>2*s*(*I*)], R1 (*all*)	0.0266	0.0335
*w*R2 [*I*>2*s*(*I*)], *w*R2 (*all*)	0.0539	0.0844
CCDC no.	2,420,972	2,420,971

## Data Availability

The data are contained in this article. Further inquiries can be directed to the corresponding author.

## Supplementary Materials

The following supporting information can be downloaded at: https://www.mdpi.com/article/10.3390/ijms26115289/s1.
