# Supplementary material for: Structural, Computational, and Biomolecular Interaction Study of Europium(III) and Iron(III) Complexes with Pyridoxal-Semicarbazone Ligand
Source: Int J Mol Sci. 2025 May 30;26(11):5289. doi: 10.3390/ijms26115289 (PMC12155188; doi:10.3390/ijms26115289)
Supplement: Supplementary file 1 [file ijms-26-05289-s001.zip › ijms-3660659-supplementary.pdf]

Supplementary Information

Structural, Computational, and Biomolecular Interaction Study of Europium(III) and Iron(III) Complexes with Pyridoxal-Semicarbazone Ligand

Violeta Jevtovic [1], Stefan Perendija [2], Aljazi Abdullah Alrashidi [1], Maha Awjan Alreshidi [1], Elham A. Alzahrani [1], Odeh A. O. Alshammari [1], Mostafa Aly Hussien [3], Jasmina Dimitrić Marković [2] and Dušan Dimić [2,*]

[1] Department of Chemistry, College of Science, University of Ha'il, Ha'il 81451, Saudi Arabia
[2] Faculty of Physical Chemistry, University of Belgrade, Studentski Trg 12-16, 11158 Belgrade, Serbia
[3] Department of Chemistry, Faculty of Science, King Abdul Aziz University, Jeddah 21589, Saudi Arabia
* Correspondence: ddimic@ffh.bg.ac.rs

Table S1. Crystallographic and optimized (at B3LYP/6-31+G(d,p)(H,C,N,O)/def2-TZVP(Eu) level of theory) bond lengths of Eu-PLSC (in Å).

Bond	Experimental bond length	Optimized bond length
Eu(1)-O(4)	2.2242(12)	2.226
Eu(1)-O(1)	2.2817(13)	2.308
Eu(1)-O(2)	2.4178(13)	2.487
Eu(1)-O(5)	2.4247(12)	2.442
Eu(1)-O(10)	2.4329(13)	2.537
Eu(1)-O(8)	2.5781(13)	2.478
Eu(1)-N(2)	2.6009(15)	2.789
Eu(1)-N(6)	2.6236(15)	2.669
Eu(1)-O(7)	2.6256(12)	2.447
Eu(1)-N(9)	2.9954(14)	2.902
Eu(1)-C(7)	3.2633(18)	3.364
O(1)-C(1)	1.295(2)	1.275
O(2)-C(7)	1.245(2)	1.237
O(3)-C(9)	1.418(2)	1.413
O(4)-C(11)	1.298(2)	1.305
O(5)-C(17)	1.248(2)	1.245
O(6)-C(19)	1.426(2)	1.413
O(7)-N(9)	1.280(2)	1.289
O(8)-N(9)	1.2702(19)	1.284
O(9)-N(9)	1.2220(19)	1.212
N(1)-C(2)	1.340(2)	1.341
N(1)-C(3)	1.350(2)	1.358
N(2)-C(6)	1.289(2)	1.292
N(2)-N(3)	1.372(2)	1.359
N(3)-C(7)	1.369(2)	1.384
N(4)-C(7)	1.335(2)	1.352
N(5)-C(12)	1.326(2)	1.323
N(5)-C(13)	1.362(2)	1.351
N(6)-C(16)	1.295(2)	1.299
N(6)-N(7)	1.385(2)	1.381
N(7)-C(17)	1.365(2)	1.365
N(8)-C(17)	1.335(2)	1.360
C(1)-C(2)	1.419(2)	1.435
C(1)-C(5)	1.422(2)	1.438
C(2)-C(8)	1.489(3)	1.491
C(3)-C(4)	1.371(3)	1.379
C(4)-C(5)	1.418(2)	1.420
C(4)-C(9)	1.519(3)	1.528
C(5)-C(6)	1.454(2)	1.453
C(11)-C(15)	1.409(2)	1.424
C(11)-C(12)	1.435(2)	1.434

C(12)-C(18)	1.499(3)	1.506
C(13)-C(14)	1.372(2)	1.384
C(14)-C(15)	1.424(2)	1.427
C(14)-C(19)	1.514(2)	1.524
C(15)-C(16)	1.453(2)	1.443
R		0.996
MAE (Å)		0.027

Table S2. Crystallographic and optimized (at B3LYP/6-31+G(d,p)(H,C,N,O)/def2-TZVP(Eu) level of theory) bond angles of Eu-PLSC (in °).

Angle	Experimental bond angle	Theoretical bond angle
O(4)-Eu(1)-O(1)	88.48(5)	93.30
O(4)-Eu(1)-O(2)	78.43(5)	76.42
O(1)-Eu(1)-O(2)	132.25(4)	126.49
O(4)-Eu(1)-O(5)	131.72(4)	130.11
O(1)-Eu(1)-O(5)	80.06(5)	81.50
O(2)-Eu(1)-O(5)	140.62(4)	143.61
O(4)-Eu(1)-O(10)	85.23(5)	80.91
O(1)-Eu(1)-O(10)	148.18(5)	157.62
O(2)-Eu(1)-O(10)	76.75(5)	73.39
O(5)-Eu(1)-O(10)	81.07(5)	85.79
O(4)-Eu(1)-O(8)	149.75(5)	138.00
O(1)-Eu(1)-O(8)	120.53(4)	127.77
O(2)-Eu(1)-O(8)	75.44(4)	72.02
O(5)-Eu(1)-O(8)	67.36(4)	72.08
O(10)-Eu(1)-O(8)	74.23(4)	64.09
O(4)-Eu(1)-N(2)	77.41(5)	78.51
O(1)-Eu(1)-N(2)	68.75(5)	65.44
O(2)-Eu(1)-N(2)	63.62(4)	61.04
O(5)-Eu(1)-N(2)	137.12(4)	138.42
O(10)-Eu(1)-N(2)	139.03(5)	133.12
O(8)-Eu(1)-N(2)	103.80(4)	108.32
O(4)-Eu(1)-N(6)	68.65(5)	67.61
O(1)-Eu(1)-N(6)	72.01(4)	84.28
O(2)-Eu(1)-N(6)	138.91(4)	134.02
O(5)-Eu(1)-N(6)	63.18(4)	62.50
O(10)-Eu(1)-N(6)	76.66(5)	73.50
O(8)-Eu(1)-N(6)	125.38(4)	118.82
N(2)-Eu(1)-N(6)	127.96(5)	132.86
O(4)-Eu(1)-O(7)	148.21(4)	145.86
O(1)-Eu(1)-O(7)	74.70(4)	80.74
O(2)-Eu(1)-O(7)	92.98(4)	80.37
O(5)-Eu(1)-O(7)	72.48(4)	82.53

O(10)-Eu(1)-O(7)	122.90(4)	115.85
O(8)-Eu(1)-O(7)	49.08(4)	52.26
N(2)-Eu(1)-O(7)	71.43(4)	68.40
N(6)-Eu(1)-O(7)	127.85(4)	143.62
O(4)-Eu(1)-N(9)	165.77(4)	150.71
O(1)-Eu(1)-N(9)	95.96(4)	104.71
O(2)-Eu(1)-N(9)	88.66(4)	74.31
O(5)-Eu(1)-N(9)	62.48(4)	76.20
O(10)-Eu(1)-N(9)	97.72(4)	89.91
O(8)-Eu(1)-N(9)	24.93(4)	26.12
N(2)-Eu(1)-N(9)	91.58(4)	88.09
N(6)-Eu(1)-N(9)	125.58(4)	136.06
O(7)-Eu(1)-N(9)	25.24(4)	26.15
O(4)-Eu(1)-C(7)	72.67(4)	73.21
O(1)-Eu(1)-C(7)	113.63(4)	109.26
O(2)-Eu(1)-C(7)	18.73(4)	17.33
O(5)-Eu(1)-C(7)	153.98(4)	154.87
O(10)-Eu(1)-C(7)	94.14(5)	89.88
O(8)-Eu(1)-C(7)	86.69(4)	83.76
N(2)-Eu(1)-C(7)	45.36(4)	43.87
N(6)-Eu(1)-C(7)	140.77(4)	139.23
O(7)-Eu(1)-C(7)	89.38(4)	77.15
N(9)-Eu(1)-C(7)	93.20(4)	79.05
C(1)-O(1)-Eu(1)	137.72(11)	147.28
C(7)-O(2)-Eu(1)	122.69(11)	125.84
C(11)-O(4)-Eu(1)	147.45(12)	149.43
C(17)-O(5)-Eu(1)	122.75(11)	124.45
N(9)-O(7)-Eu(1)	93.76(9)	97.08
N(9)-O(8)-Eu(1)	96.24(10)	95.74
C(2)-N(1)-C(3)	124.58(17)	124.85
C(6)-N(2)-N(3)	114.87(15)	116.17
C(6)-N(2)-Eu(1)	132.52(12)	131.90
N(3)-N(2)-Eu(1)	112.52(10)	111.88
C(7)-N(3)-N(2)	117.46(15)	118.68
C(12)-N(5)-C(13)	119.27(15)	119.39
C(16)-N(6)-N(7)	114.38(15)	115.50
C(16)-N(6)-Eu(1)	132.45(12)	131.51
N(7)-N(6)-Eu(1)	112.79(10)	112.91
C(17)-N(7)-N(6)	117.56(15)	117.86
O(9)-N(9)-O(8)	122.40(15)	122.62
O(9)-N(9)-O(7)	121.69(15)	122.47
O(8)-N(9)-O(7)	115.90(14)	114.90
O(9)-N(9)-Eu(1)	160.06(12)	178.63
O(8)-N(9)-Eu(1)	58.82(8)	58.14
O(7)-N(9)-Eu(1)	61.00(8)	56.78

O(1)-C(1)-C(2)	117.83(16)	117.69
O(1)-C(1)-C(5)	123.83(16)	124.94
C(2)-C(1)-C(5)	118.31(15)	117.36
N(1)-C(2)-C(1)	118.42(17)	118.98
N(1)-C(2)-C(8)	119.66(17)	120.26
C(1)-C(2)-C(8)	121.92(16)	120.76
N(1)-C(3)-C(4)	119.95(17)	119.36
C(3)-C(4)-C(5)	118.95(17)	119.65
C(3)-C(4)-C(9)	119.17(16)	117.06
C(5)-C(4)-C(9)	121.89(16)	123.26
C(4)-C(5)-C(1)	119.59(16)	119.74
C(4)-C(5)-C(6)	118.62(16)	118.81
C(1)-C(5)-C(6)	121.78(15)	121.45
N(2)-C(6)-C(5)	123.84(16)	125.00
O(2)-C(7)-N(4)	123.39(16)	122.73
O(2)-C(7)-N(3)	120.94(16)	121.82
N(4)-C(7)-N(3)	115.66(16)	115.44
O(2)-C(7)-Eu(1)	38.57(8)	36.82
N(4)-C(7)-Eu(1)	160.99(12)	159.09
N(3)-C(7)-Eu(1)	82.65(10)	85.19
O(3)-C(9)-C(4)	112.64(15)	112.86
O(4)-C(11)-C(15)	123.73(16)	123.06
O(4)-C(11)-C(12)	118.06(16)	119.42
C(15)-C(11)-C(12)	118.20(15)	117.52
N(5)-C(12)-C(11)	121.93(16)	122.56
N(5)-C(12)-C(18)	119.73(16)	118.20
C(11)-C(12)-C(18)	118.33(15)	119.24
N(5)-C(13)-C(14)	123.54(16)	123.91
C(13)-C(14)-C(15)	118.41(16)	117.90
C(13)-C(14)-C(19)	120.75(15)	119.67
C(15)-C(14)-C(19)	120.83(15)	122.43
C(11)-C(15)-C(14)	118.60(15)	118.70
C(11)-C(15)-C(16)	122.32(15)	121.56
C(14)-C(15)-C(16)	119.04(16)	119.70
N(6)-C(16)-C(15)	124.58(16)	126.07
O(5)-C(17)-N(8)	123.02(16)	121.85
O(5)-C(17)-N(7)	120.70(16)	121.14
N(8)-C(17)-N(7)	116.25(17)	116.98
O(6)-C(19)-C(14)	112.97(14)	114.19
R		0.98
MAE (°)		3.42

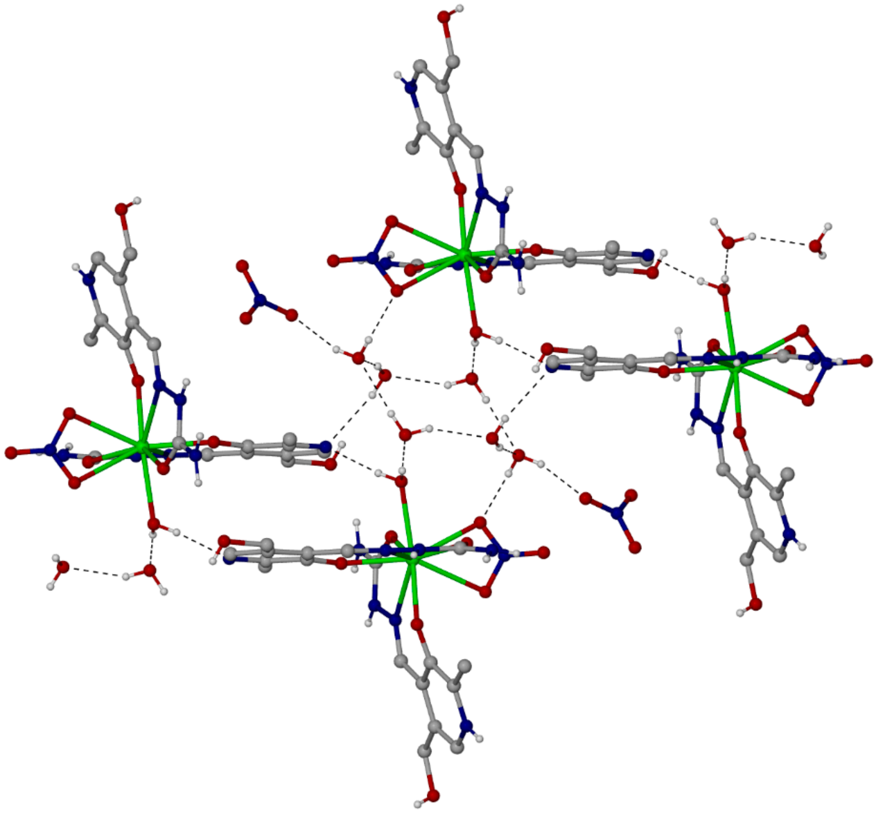

Figure S1. Hydrogen bond connectivity of the lattice water and $[NO_3]^-$ anions in the structure of $[Eu(PLSC)(PLSC\text{-}H)(NO_3)(H_2O)]\cdot[NO_3]\cdot3(H_2O)$. Hydrogen bonds are listed in Table S3.

Table S3. Hydrogen bonds for Eu-PLSC [Å and °].

**D–H···A**	**d(D–H)**	**d(H···A)**	**d(D···A)**	**∠(DHA)**
O(3)–H(3A)···O(11)	0.74(3)	2.05(3)	2.794(2)	174(3)
O(6)–H(6A)···O(1)#1	0.71(3)	2.30(3)	2.8921(19)	141(3)
O(10)–H(10A)···O(14)	0.76(3)	2.03(3)	2.789(2)	173(2)
O(10)–H(10B)···N(5)#2	0.83(3)	1.99(3)	2.813(2)	174(3)
O(14)–H(14A)···O(15)	0.77(3)	2.03(3)	2.746(2)	155(3)
O(14)–H(14B)···O(16)#3	0.79(3)	2.03(3)	2.808(2)	171(3)
O(15)–H(15A)···N(5)#4	0.74(3)	2.21(3)	2.943(2)	171(3)
O(15)–H(15B)···O(16)#5	0.77(3)	2.06(3)	2.814(2)	165(3)
O(16)–H(16A)···O(8)#5	0.77(3)	2.05(3)	2.815(2)	178(3)
O(16)–H(16B)···O(13)	0.76(3)	2.09(4)	2.796(2)	155(4)
N(1)–H(1N)···O(7)#5	0.82(2)	2.08(2)	2.809(2)	148(2)
N(3)–H(2N)···O(12)#6	0.83(2)	2.10(2)	2.902(2)	161(2)
N(4)–H(3N)···O(11)#6	0.83(3)	2.24(3)	3.018(2)	157(2)
N(4)–H(4N)···O(6)#2	0.88(3)	2.06(3)	2.910(2)	162(2)
N(7)–H(5N)···O(15)#7	0.82(3)	2.13(3)	2.926(2)	166(3)
N(8)–H(6N)···O(14)#7	0.86(3)	2.09(3)	2.937(2)	173(3)
N(8)–H(7N)···O(3)#5	0.81(3)	2.11(3)	2.873(2)	156(2)

Symmetry transformations used to generate equivalent atoms:
#1 –x+1, –y, –z+2
#2 –x+2, –y, –z+2

#3 x+1, y, z+1
#4 x, y+1, z
#5 –x+1, –y+1, –z+1
#6 x+1, y, z
#7 –x+1, –y+1, –z+2

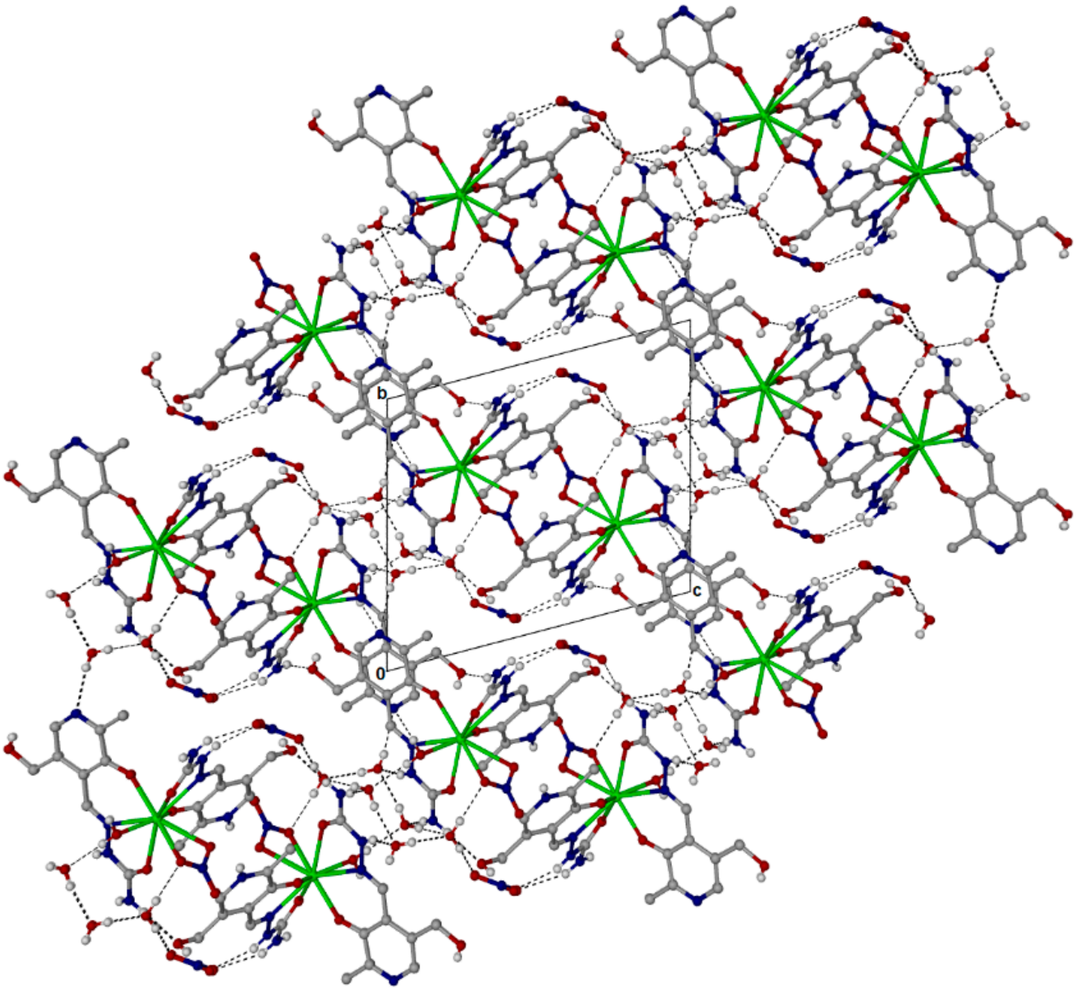

Figure S2. Cell packing of $[Eu(PLSC)(PLSC\text{-}H)(NO_3)(H_2O)]\cdot[NO_3]\cdot3(H_2O)$ as viewed down the *a* axis.

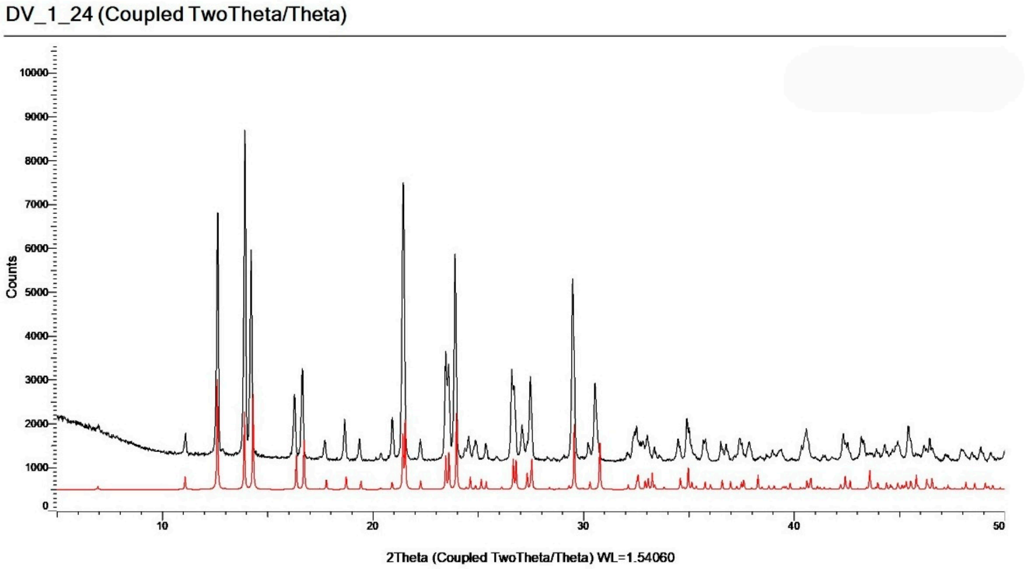

Figure S3. Experimental(black) and calculated (red) PXRD patterns for $[Fe(PLSC)Cl_2(H_2O)][Cl]$. Note: the majority of the sample appeared to be a dark red-orange powder containing a few isolated crystals. However, a PXRD of the bulk material was consistent with that calculated from the crystal structure, indicating that there was only one crystalline product.

Table S4. Crystallographic and optimized (at B3LYP/6-311++G(d,p)(H,C,N,O,Cl)/def2-TZVP(Fe) level of theory) bond lengths of Fe-PLSC (in Å).

Bond	Experimental bond length	Theoretical bond length
Fe(1)-O(2)	1.922(3)	1.914
Fe(1)-O(1)	2.062(3)	1.978
Fe(1)-O(4)	2.109(3)	2.083
Fe(1)-N(3)	2.189(4)	1.944
Fe(1)-Cl(1)	2.2701(12)	2.250
Fe(1)-Cl(2)	2.2876(11)	2.237
O(1)-C(1)	1.251(6)	1.248
O(2)-C(7)	1.306(5)	1.275
O(3)-C(8)	1.430(5)	1.426
N(1)-C(1)	1.330(5)	1.341
N(2)-C(1)	1.356(6)	1.389
N(2)-N(3)	1.370(5)	1.383
N(3)-C(2)	1.292(5)	1.289
N(4)-C(6)	1.330(6)	1.338
N(4)-C(5)	1.343(6)	1.362
C(2)-C(3)	1.456(5)	1.448
C(3)-C(7)	1.406(6)	1.436
C(3)-C(4)	1.425(6)	1.419
C(4)-C(5)	1.380(6)	1.373

C(4)-C(8)	1.509(6)	1.512
C(6)-C(7)	1.425(5)	1.432
C(6)-C(9)	1.476(7)	1.488
R		0.99
MAE (Å)		0.023

Table S5. Crystallographic and optimized (at B3LYP/6-311++G(d,p)(H,C,N,O,Cl)/def2-TZVP(Fe) level of theory) bond angles of Fe-PLSC (in °).

Angle	Experimental bond angle	Theoretical bond angle
O(2)-Fe(1)-O(1)	156.03(12)	172.21
O(2)-Fe(1)-O(4)	87.38(13)	92.51
O(1)-Fe(1)-O(4)	84.34(13)	80.73
O(2)-Fe(1)-N(3)	81.88(12)	92.51
O(1)-Fe(1)-N(3)	74.98(12)	80.73
O(4)-Fe(1)-N(3)	84.65(14)	84.78
O(2)-Fe(1)-Cl(1)	103.49(10)	90.92
O(1)-Fe(1)-Cl(1)	98.38(9)	95.17
O(4)-Fe(1)-Cl(1)	86.26(11)	86.60
N(3)-Fe(1)-Cl(1)	169.23(10)	170.52
O(2)-Fe(1)-Cl(2)	93.83(9)	90.82
O(1)-Fe(1)-Cl(2)	93.29(9)	93.16
O(4)-Fe(1)-Cl(2)	176.54(11)	175.03
N(3)-Fe(1)-Cl(2)	92.31(10)	91.01
Cl(1)-Fe(1)-Cl(2)	96.59(4)	97.78
C(1)-O(1)-Fe(1)	117.8(3)	111.60
C(7)-O(2)-Fe(1)	134.7(3)	124.66
C(1)-N(2)-N(3)	115.8(4)	110.56
C(2)-N(3)-N(2)	117.4(3)	118.00
C(2)-N(3)-Fe(1)	131.6(3)	128.15
N(2)-N(3)-Fe(1)	111.0(3)	113.21
C(6)-N(4)-C(5)	124.8(4)	124.63
O(1)-C(1)-N(1)	122.9(4)	122.20
O(1)-C(1)-N(2)	120.4(4)	121.66
N(1)-C(1)-N(2)	116.7(4)	116.14
N(3)-C(2)-C(3)	121.1(4)	122.40
C(7)-C(3)-C(4)	120.0(4)	119.46
C(7)-C(3)-C(2)	121.8(4)	121.38
C(4)-C(3)-C(2)	118.2(4)	119.14
C(5)-C(4)-C(3)	118.2(4)	119.78
C(5)-C(4)-C(8)	118.5(4)	117.87
C(3)-C(4)-C(8)	123.3(4)	122.34
N(4)-C(5)-C(4)	120.0(4)	119.48
N(4)-C(6)-C(7)	118.5(4)	118.96

N(4)-C(6)-C(9)	119.3(4)	120.50
C(7)-C(6)-C(9)	122.2(4)	120.53
O(2)-C(7)-C(3)	125.2(3)	127.18
O(2)-C(7)-C(6)	116.6(4)	115.15
C(3)-C(7)-C(6)	118.2(4)	117.66
O(3)-C(8)-C(4)	108.3(4)	108.49
R		0.98
MAE (°)		2.77

Table S6. Hydrogen bonds for Fe-PLSC [Å and °].

D–H···A	d(D–H)	d(H···A)	d(D···A)	∠(DHA)
O(3)–H(3)···Cl(3)#1	0.86(9)	2.43(9)	3.273(4)	167(7)
O(4)–H(4A)···Cl(3)	0.83(9)	2.21(9)	3.020(3)	167(8)
O(4)–H(4B)···Cl(3)#2	0.86(8)	2.21(8)	3.065(3)	175(7)
N(1)–H(1N)···Cl(3)#3	0.86(8)	2.41(8)	3.228(4)	159(6)
N(1)–H(2N)···Cl(3)#4	0.83(6)	2.85(6)	3.531(5)	141(4)
N(2)–H(3N)···Cl(3)#4	0.84(7)	2.39(7)	3.170(4)	156(6)
N(4)–H(4N)···Cl(2)#5	0.92(7)	2.37(7)	3.142(4)	141(6)

Symmetry transformations used to generate equivalent atoms:
#1 x–1, y, z
#2 –x+2, y–½, –z+3⁄2
#3 x, y–1, z
#4 –x+1, y–½, –z+3⁄2
#5 x, y+1, z

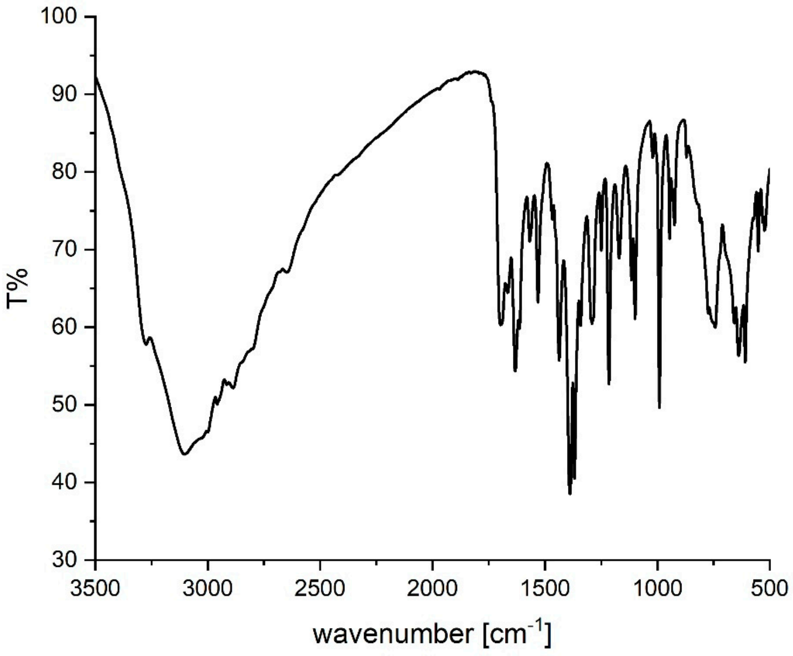

Figure S4. FTIR spectrum of Eu-PLSC.

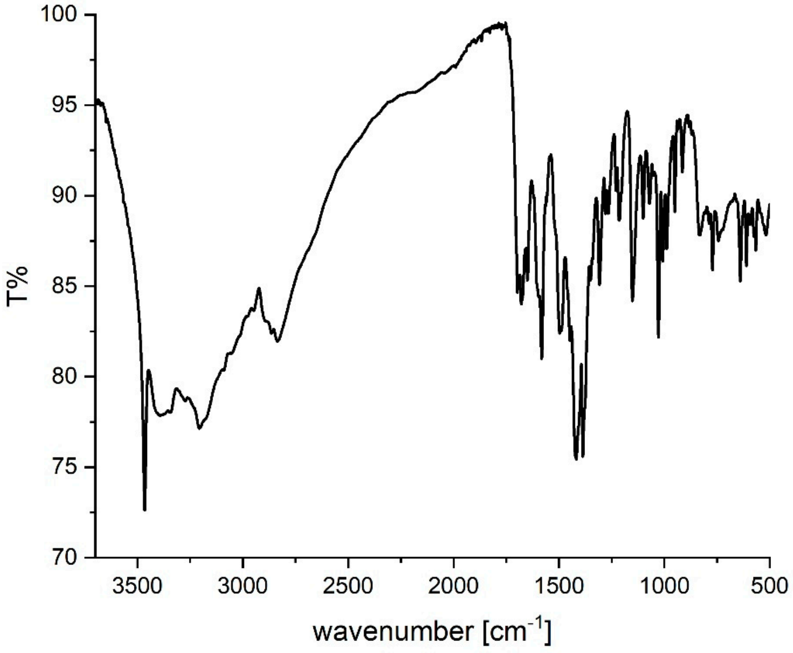

Figure S5. FTIR spectrum of Fe-PLSC.

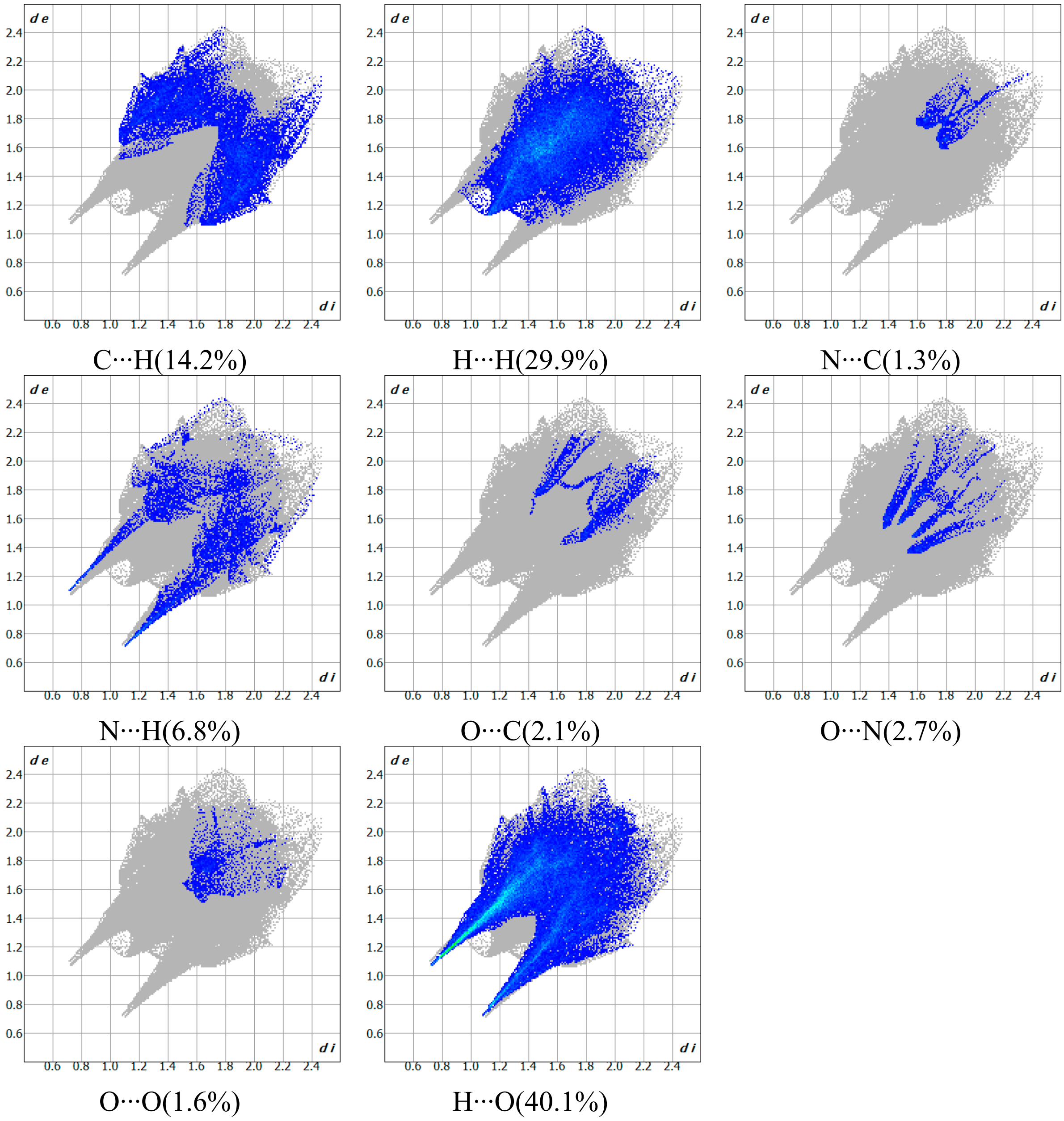

Figure S6. The most numerous contacts in the crystallographic structure of Eu-PLSC.

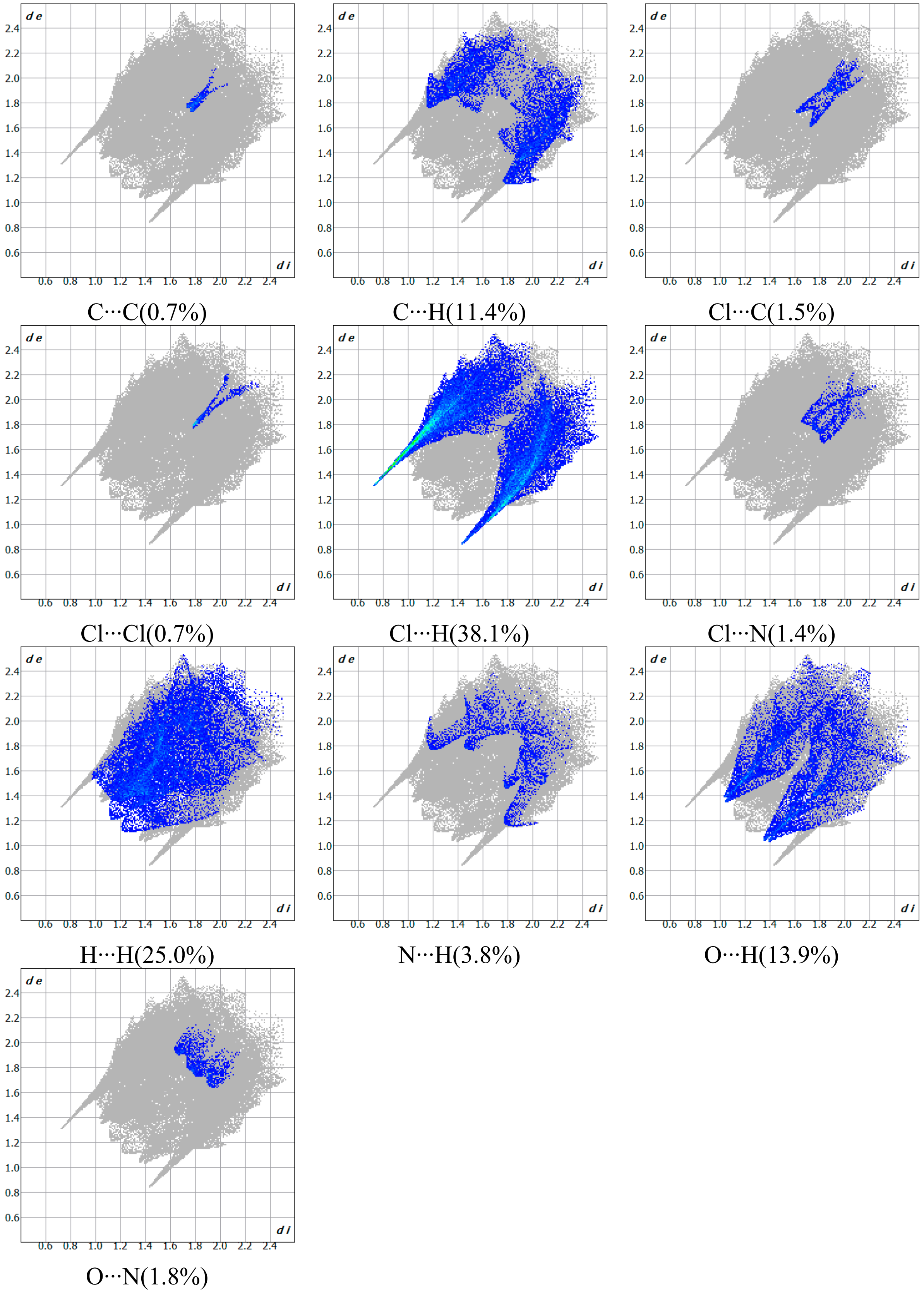

Figure S7. The most numerous contacts in the crystallographic structure of Fe-PLSC.

Table S7. The calculated Bond Critical Points (BCP) properties: the electron density (ρ(r)) and its Laplacian ($\nabla^2\rho(r)$); the Lagrangian kinetic electron density (G(r)) and the potential electron density (V(r)); the density of the total energy of electrons (H(r)) – Cremer-Kraka electronic energy density; the interatomic bond energy, E_{bond},

Bond	ρ(r) [a.u.]	$\nabla^2\rho(r)$ [a.u.]	G(r) [kJ mol^{-1}]	V(r) [kJ mol^{-1}]	H(r) [kJ mol^{-1}]	-G(r)/V(r)	E_{bond} [kJ mol^{-1}]
Eu-PLSC							
Eu−O1	0.058	0.261	170.9	-170.5	0.4	1.0	-85.2
Eu−O2	0.039	0.164	102.9	-98.4	4.5	1.0	-49.2
Eu−O4	0.073	0.328	228.4	-241.2	-12.8	0.9	-120.6
Eu−O5	0.045	0.186	119.5	-116.9	2.6	1.0	-58.5
Eu−O7	0.049	0.182	122.5	-125.7	-3.2	1.0	-62.9
Eu−O8	0.045	0.167	111.7	-113.8	-2.1	1.0	-56.9
Eu−O10	0.037	0.145	91.5	-87.9	3.6	1.0	-44.0
Eu−N2	0.023	0.085	50.5	-45.4	5.1	1.1	-22.7
Eu−N6	0.031	0.109	68.1	-64.9	3.2	1.0	-32.4
O3···H	0.017	0.076	43.0	-36.0	7.0	1.2	-18.0
O1···N2	0.016	0.050	30.8	-29.0	1.9	1.1	-14.5
N3···HCH_2	0.003	0.009	4.8	-3.6	1.3	1.4	-1.8
O8···O11	0.017	0.078	45.2	-39.4	5.9	1.1	-19.7
C19···H	0.011	0.051	25.8	-17.9	7.9	1.4	-8.9
Fe-PLSC							
Fe−O1	0.077	0.466	317.4	-329.1	-11.7	1.0	-164.6
Fe−O2	0.091	0.541	376.7	-398.2	-21.5	0.9	-199.1
Fe−O4	0.059	0.323	219.7	-227.5	-7.9	1.0	-113.8
Fe−N3	0.101	0.471	369.4	-429.6	-60.3	0.9	-214.8
Fe−Cl1	0.077	0.199	187.5	-244.1	-56.6	0.8	-122.0
Fe−Cl2	0.080	0.187	181.8	-240.7	-58.9	0.8	-120.4
HOH···Cl3	0.028	0.064	44.1	-46.4	-2.4	0.9	-23.2
N2H···Cl3	0.054	0.066	80.4	-117.1	-36.7	0.7	-58.6
O3···H	0.013	0.045	25.5	-21.5	4.0	1.2	-10.8

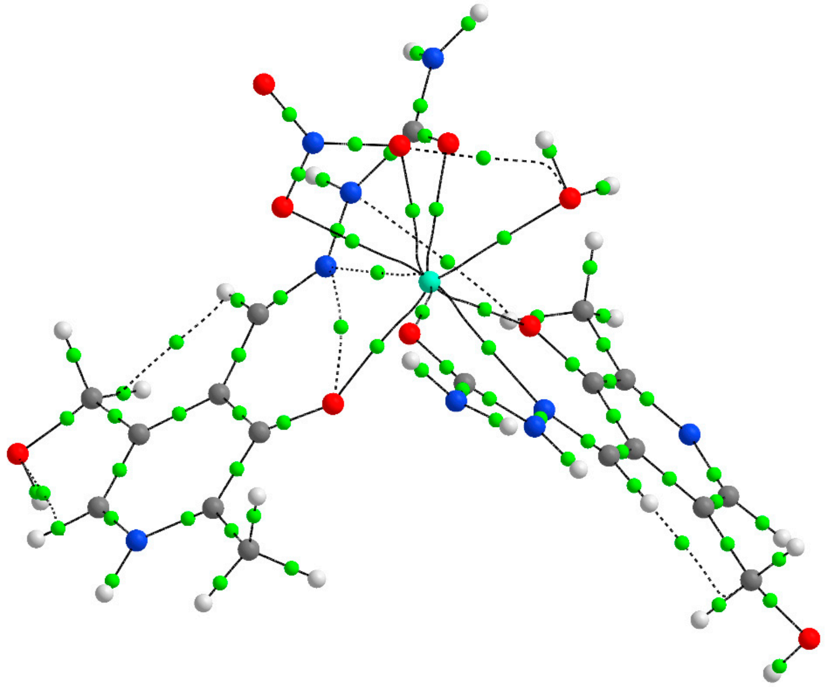

Figure S8. Representation of the Bond Critical Points in the optimized structure of Eu-PLSC.

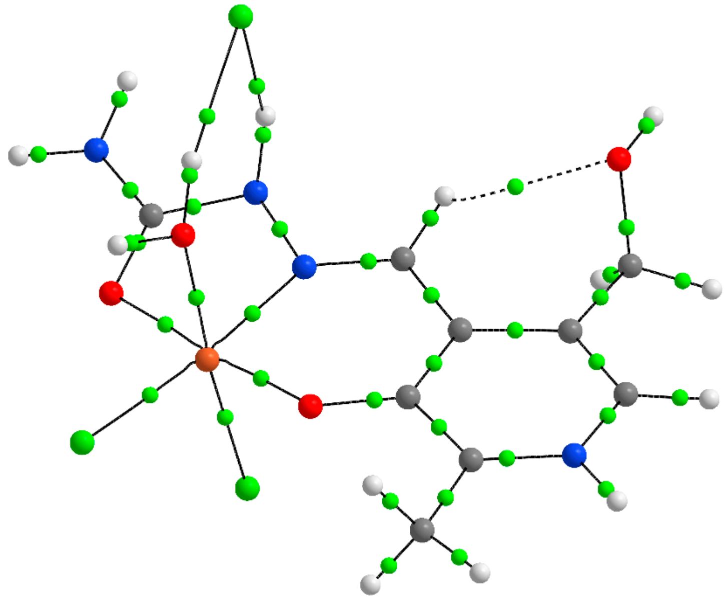

Figure S9. Representation of the Bond Critical Points in the optimized structure of Fe-PLSC.

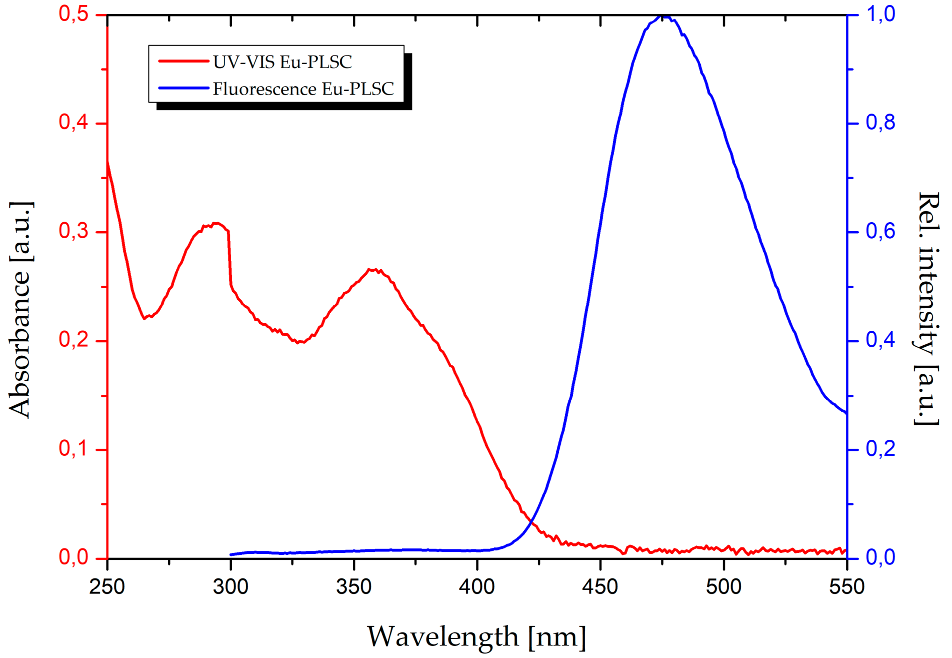

Figure S10. UV-VIS and fluorescence (excitation wavelength 280 nm) spectra of **Eu-PLSC**.

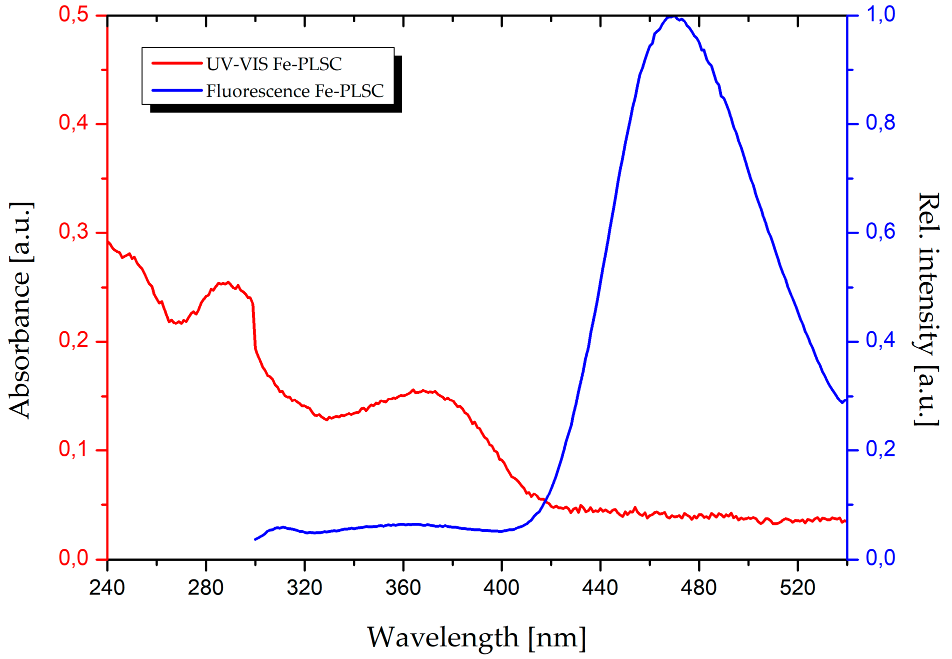

Figure S11. UV-VIS and fluorescence (excitation wavelength 280 nm) spectra of **Fe-PLSC**.

Table S8. The interactions formed between biomolecules and complexes in the molecular docking simulations.

Eu-PLSC and HSA (PDB ID: 6EZQ)		
Eu-PLSC group	Interaction	Amino acid (group)
PLSC1 ($-CH_2-OH$ group)	O−H···N classic hydrogen bond	Asn429
PLSC2 (−NH−)	N−H···O−H classic hydrogen bond	Tyr452
PLSC2	π···LP	Tyr452 (benzene-ring)
PLSC1 ($-NH_2$)	N−H···O classic hydrogen bond	Asp451 (amide bond)
PLSC1 ($-NH_2$)	N−H···O classic hydrogen bond	Asp451 (carboxylic group)
Nitrate ion	$N^+\cdots O^-$ attractive charge	Asp451
PLSC2 (pyridine-ring)	C−H···O carbon-hydrogen bond	Glu188
PLSC2 ($-CH_2-OH$ group)	O−H···N classic hydrogen bond	Glu292
Nitrate ion	$O^-\cdots O^-$ unfavourable negative-negative	Glu292
Nitrate ion	$N^+\cdots O^-$ attractive charge	Glu292
PLSC2 (SC group)	$N^+\cdots O^-$ attractive charge	Glu292 (carboxylic group)
Nitrate ion	$N^+\cdots O^-$ attractive charge	Lys195
Nitrate ion	$N^+\cdots O^-$ attractive charge	Lys195
**Fe-PLSC and HSA (PDB ID: 6EZQ)**		
Eu-PLSC group	Interaction	Amino acid (group)
PLSC (pyridine-ring)	−H···O classic hydrogen bond	Asp451
PLSC ($-CH_2-OH$ group)	−O···H classic hydrogen bond	Cys448
PLSC ($-CH_2-OH$ group)	−H···O classic hydrogen bond	Lys444
PLSC (SC ammonium cation)	$-N^+-H\cdots O$ salt bridge hydrogen bond	Glu294 (carboxylic group)
PLSC ($-NH_2$)	N−H···O classic hydrogen bond	Glu294 (carboxylic group)
PLSC (SC ammonium cation)	$-\ HN^+\cdots N^+$ unfavourable positive-positive	Arg222
PLSC (SC ammonium cation)	$-\ H_2N^+\cdots N^+$ unfavourable positive-positive	Arg218
PLSC ($-NH_2$)	N−H···O classic hydrogen bond	Glu292 (carboxylic group)
Water	H···O classic hydrogen bond	Val293
**Eu-PLSC and DNA (PDB ID: 1BNA)**		
Eu-PLSC group	Interaction	Nucleotide (group)
PLSC1 (SC ammonium cation)	H···O−P salt bridge hydrogen bond	A:Thy8 (phosphate group)
PLSC2 (SC ammonium cation)	C−H···O carbon-hydrogen bond	A:Thy8 (Sugar-ring)
PLSC1 (SC ammonium cation)	$-N^+-H\cdots O$ salt bridge hydrogen bond	A:Thy8 (Sugar-ring)
PLSC2 (SC group)	C−H···O carbon-hydrogen bond	A:Thy7 (Sugar-ring)
Nitrate ion	$O^-\cdots O^-$ unfavourable negative-negative	B:Cyt21 (phosphate group)
**Fe-PLSC intercalation in DNA (PDB ID: 454D)**		
Fe-PLSC group	Interaction	Nucleotide (group)
PLSC ($-CH_3$)	π···σ	B:Cyt13 (Pyrimidine-ring)
PLSC	van der Walls	A:Gua4 (Pyrimidine-ring)
Water1	Unfavorable donor-donor	B: Gua12 (Pyrimidine-ring)
Water1	-O−H···N classic hydrogen bond	B: Gua12 (Pyrimidine-ring)
PLSC ($-CH_3$)	π···σ	B: Gua12 (Pyrimidine-ring)
PSLC	van der Walls	B: Ade14 (Pyrimidine-ring)
Water2	-O−H···N classic hydrogen bond	A:Cyt5 (Pyrimidine-ring)
PSLC	van der Walls	A: Ade7 (Pyrimidine-ring)
PLSC (SC ammonium cation)	−H···N classic hydrogen bond	A: Ade6 (Pyrimidine-ring)
PLSC (SC group)	−H···N classic hydrogen bond	A: Ade6 (Pyrimidine-ring)
